# Neurocomputational mechanisms underlying fear-biased adaptation learning in changing environments

**DOI:** 10.1371/journal.pbio.3001724

**Published:** 2023-05-01

**Authors:** Zhihao Wang, Tian Nan, Katharina S. Goerlich, Yiman Li, André Aleman, Yuejia Luo, Pengfei Xu

**Affiliations:** 1 Beijing Key Laboratory of Applied Experimental Psychology, National Demonstration Center for Experimental Psychology Education (BNU), Faculty of Psychology, Beijing Normal University, Beijing, China; 2 CNRS—Centre d’Economie de la Sorbonne, Panthéon-Sorbonne University, France; 3 School of Psychology, Sichuan Center of Applied Psychology, Chengdu Medical College, Chengdu, China; 4 University of Groningen, Department of Biomedical Sciences of Cells & Systems, Section Cognitive Neuroscience, University Medical Center Groningen, Groningen, the Netherlands; 5 Shenzhen Key Laboratory of Affective and Social Neuroscience, Magnetic Resonance Imaging, Center for Brain Disorders and Cognitive Sciences, Shenzhen University, Shenzhen, China; 6 The State Key Lab of Cognitive and Learning, Faculty of Psychology, Beijing Normal University, Beijing, China; 7 Center for Neuroimaging, Shenzhen Institute of Neuroscience, Shenzhen, China; Oxford University, UNITED KINGDOM

## Abstract

Humans are able to adapt to the fast-changing world by estimating statistical regularities of the environment. Although fear can profoundly impact adaptive behaviors, the computational and neural mechanisms underlying this phenomenon remain elusive. Here, we conducted a behavioral experiment (*n* = 21) and a functional magnetic resonance imaging experiment (*n* = 37) with a novel cue-biased adaptation learning task, during which we simultaneously manipulated emotional valence (fearful/neutral expressions of the cue) and environmental volatility (frequent/infrequent reversals of reward probabilities). Across 2 experiments, computational modeling consistently revealed a higher learning rate for the environment with frequent versus infrequent reversals following neutral cues. In contrast, this flexible adjustment was absent in the environment with fearful cues, suggesting a suppressive role of fear in adaptation to environmental volatility. This suppressive effect was underpinned by activity of the ventral striatum, hippocampus, and dorsal anterior cingulate cortex (dACC) as well as increased functional connectivity between the dACC and temporal-parietal junction (TPJ) for fear with environmental volatility. Dynamic causal modeling identified that the driving effect was located in the TPJ and was associated with dACC activation, suggesting that the suppression of fear on adaptive behaviors occurs at the early stage of bottom-up processing. These findings provide a neuro-computational account of how fear interferes with adaptation to volatility during dynamic environments.

## Abstract 2

This study shows that adaptation to rapidly changing environments is suppressed by fear at the early stage of bottom-up processing of fearful information, providing a neuro-computational account of how fear interferes with adaptive behavior in response to dynamic situations.

## Introduction

Humans and animals are able to adapt to the fast-changing world. Fear, the most studied emotion despite differences regarding its definition and measurement [1–4], profoundly influences adaptive behavior [5,6]. Although adaptation to dynamic environments is a key form of behavioral flexibility, how fear affects adaptation to dynamic environments remains unclear. Evolutionarily, fear acts as an alerting signal for self-protection [7]. A rich literature illustrates beneficial effects of fear on flexibility [2,6,8,9]. Thus, elicitation of fearful signals may facilitate adaptation to dynamic environments. In contrast, when dominating consciousness, fear can disrupt systems supporting flexible behavior [5,10], and lesions of fear circuits may even facilitate flexible performance [11]. Consequently, the conscious experience of fear may suppress adaptation to changing environments.

Adaptation to dynamic environments depends on the internal representation of uncertainties [12], which can be classified into 2 types: expected uncertainty and unexpected uncertainty [12–14]. The former refers to noise in the action–outcome association, for example, when choosing the correct option occasionally results in an undesirable outcome. The latter is characterized as volatility or the frequency at which action–outcome contingencies change. For example, after the switch of the action–outcome association, an action that was primarily associated with a given outcome becomes predominantly associated with another. Optimal adaptive behavior depends on accurate identification of the source of uncertainty [14–17]. More specifically, if unexpected outcomes are caused by noise, the current action is optimally guided by averaging previous observations. Instead, if unexpected outcomes result from environmental volatility, only recent outcomes are necessary to determine the present action. According to reinforcement learning theory [18], human learners can adapt to changing environments, exhibiting a higher learning rate in the volatile relative to stable environment [14,16]. The Bayesian learner has also been demonstrated to dynamically track environmental volatility with optimal performance [14,17]. Significant steps forward in uncertainty-related studies point toward the relevance of affective representations [19–21]. Emotional responses contribute to adaptation to volatility [14], and failure to adapt to environmental volatility has been identified as a major contributor to affective disorders [17,22–24]. Animal studies demonstrated a facilitating role of serotonin neurons in flexible adaptation to volatility [19] and observed that levels of the neurotransmitter serotonin modulated fear processing [25], suggesting an important link between fear and adaptation to volatility.

Previous neuroimaging studies on adaptation to volatility have implicated a distributed brain system involving the dorsal anterior cingulate cortex (dACC) that represents subjective estimation of environmental volatility [14,26], the amygdala and the hippocampus (HI) that encode valance-dependent conditioning and storage [13,27,28], and the ventral striatum (VS) and the orbitofrontal cortex (OFC) process uncertain-related value information [27,29]. For example, Behrens and colleagues (2007) has observed that the dACC is involved in the estimation of reward structure for decisions to be made effectively. Interestingly, these brain areas are also of importance for emotional processing, in particular fearful experience. Signals from the dACC have been linked to the processing of emotion–cognition integration (facilitation and inhibition) [30]. As a fear circuitry hub, the amygdala has been shown to influence interactions between fearful reactions and executive functions [30]. The HI has long been considered crucial for fear memory [6,31]. The VS and OFC have been responsible for affective computing from learning signals [32,33]. Therefore, these candidate brain areas may influence the interplay between fear and adaptation to volatility.

This study aimed to examine the effect of fear on adaptation to volatility and its underlying neuro-computational mechanisms. From an evolutionary perspective, it can be hypothesized that fear would facilitate adaptation to volatility learning. Alternatively, fear could prevent adaptation to volatility learning due to the cognitive cost of fear processing and a flight response induced by fearful stimuli with which the individual seeks to escape the volatile environment. The region of interests (ROIs) in the fear-biased adaption to volatility included the dACC, amygdala, HI, VS, and OFC. To test these hypotheses, we used computational modeling and functional magnetic resonance imaging (fMRI) in a novel cue-biased adaptation learning task. Based on the framework of the probabilistic reward reversal learning task [14,24], the current task simultaneously manipulated emotional valence of the cue (fearful/neutral facial expressions) and environmental volatility (frequent/infrequent reversals of reward probability) [16]. We also took into account individual differences in propensity for emotion processing and regulation. More specifically, this regards alexithymia, which refers to a reduced ability to identify, describe, and regulate one’s feelings [34]. Given cognitive and emotional deficits in alexithymia [35], we explored associations between alexithymia levels and fear-biased adaptation to volatility learning.

We observed a higher learning rate for the environment with frequent compared to infrequent reversals with the cue of neutral face, which was consistent with previous studies [14,17,24]. However, this pattern was absent in the fearful cues, suggesting a suppressive role of fear in adaptation to volatility and thus supporting the second hypothesis. Interestingly, this bias was stronger with higher levels of alexithymia. We also revealed the distributed neural substrates underlying computations of fear-biased adaptation to volatility learning, including integration systems, learning systems, and memory systems. In particular, the TPJ-dACC pathway was found to influence the interplay between fear and adaptation to volatility learning. It implies that the suppression of fear affects adaptive behaviors at a relatively early stage of processing bottom-up inputs.

## Results

**Fig 1:**
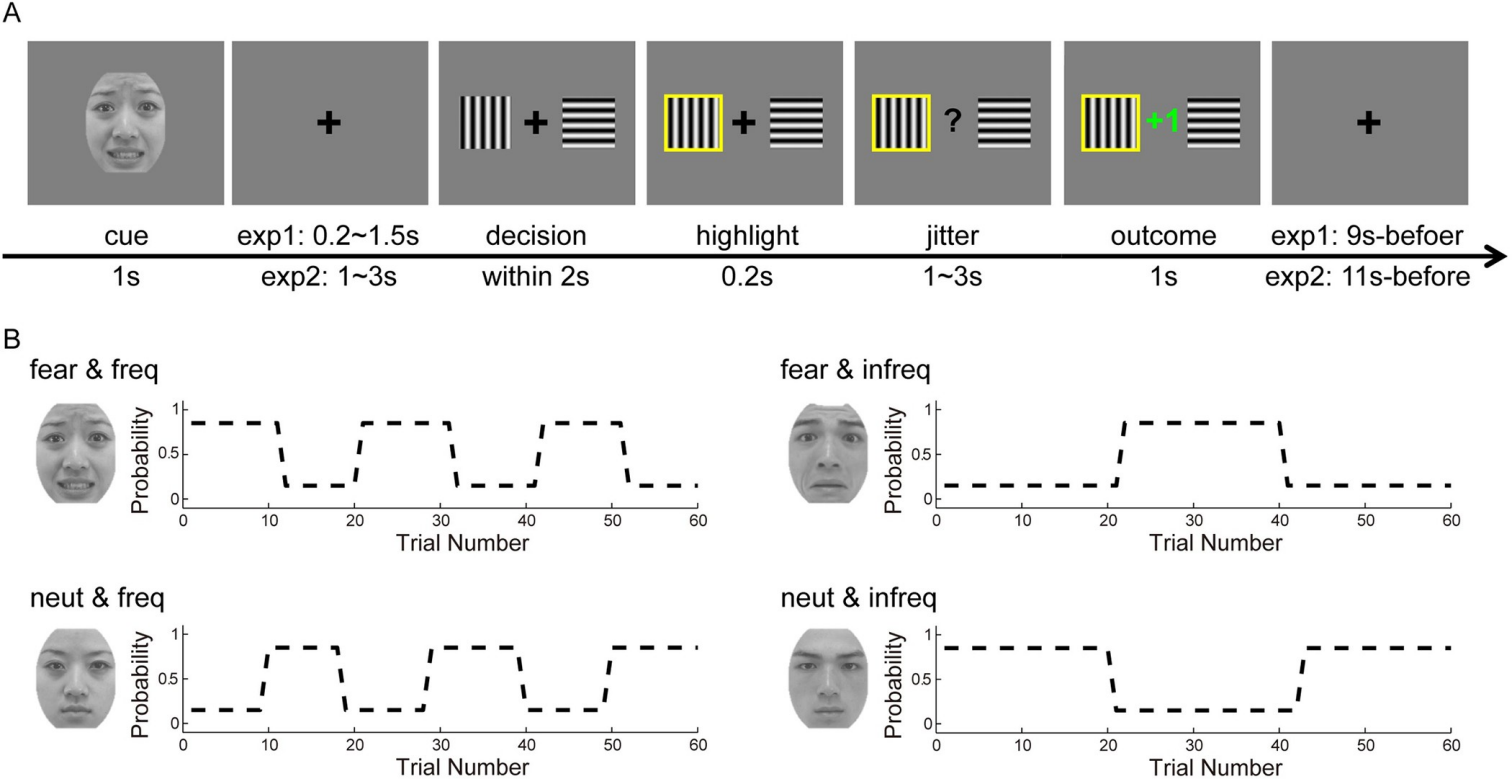
Experimental design. (A) Trial design of the fear-biased volatility learning task and (B) an example of contingency between cue and environmental volatility. These facial expressions we used as examples are free of copyright from the TFEID [74]. TFEID, Taiwanese Facial Expression Image Database.

Participants completed the cue-biased adaptation learning task (Fig 1) in Experiment 1 (exp1; *n* = 21, behavioral study) and Experiment 2 (exp2; *n* = 40, fMRI study; see Table 1 for demographic information). Using the framework of the probabilistic reward reversal learning task [14,24], we developed a 2 [emotional valence of cue: fearful/neutral expressions (fear/neut)] by 2 [environmental volatility: frequent/infrequent reversals (freq/infreq); Fig 1] within-subject design to test how fear influences adaptation to volatility.

**Table 1:** Demographics and questionnaire scores.

	exp 1 (*n* = 21)	exp 2 (*n* = 40)
Age	20.81 (1.94)	21.75 (2.13)
Gender (female)	11	18
BVAQ	122.71 (8.76)	124.70 (10.71)
BVAQ-C	74.90 (7.87)	74.10 (7.28)
BVAQ-A	47.81 (6.31)	50.60 (5.69)
MASQ-AA	21.95 (6.90)	23.03 (5.91)
MASQ-AD	63.10 (14.05)	59.90 (12.33)
MASQ-GDA	15.14 (5.63)	16.68 (5.04)
MASQ-GDD	18.14 (6.22)	18.73 (7.34)

Descriptive data are presented as mean (SD).

BVAQ, the Bermond–Vorst Alexithymia Questionnaire; BVAQ-C, the cognitive dimension of BVAQ; BVAQ-A, the affective dimension of BVAQ; MASQ-AA, anxious arousal of the Mood and Anxiety Symptoms Questionnaire; MASQ-AD, anhedonia depression; GDA, general distress: anxiety; GDD, general distress: depression.

### Manipulation checks

**Fig 2:**
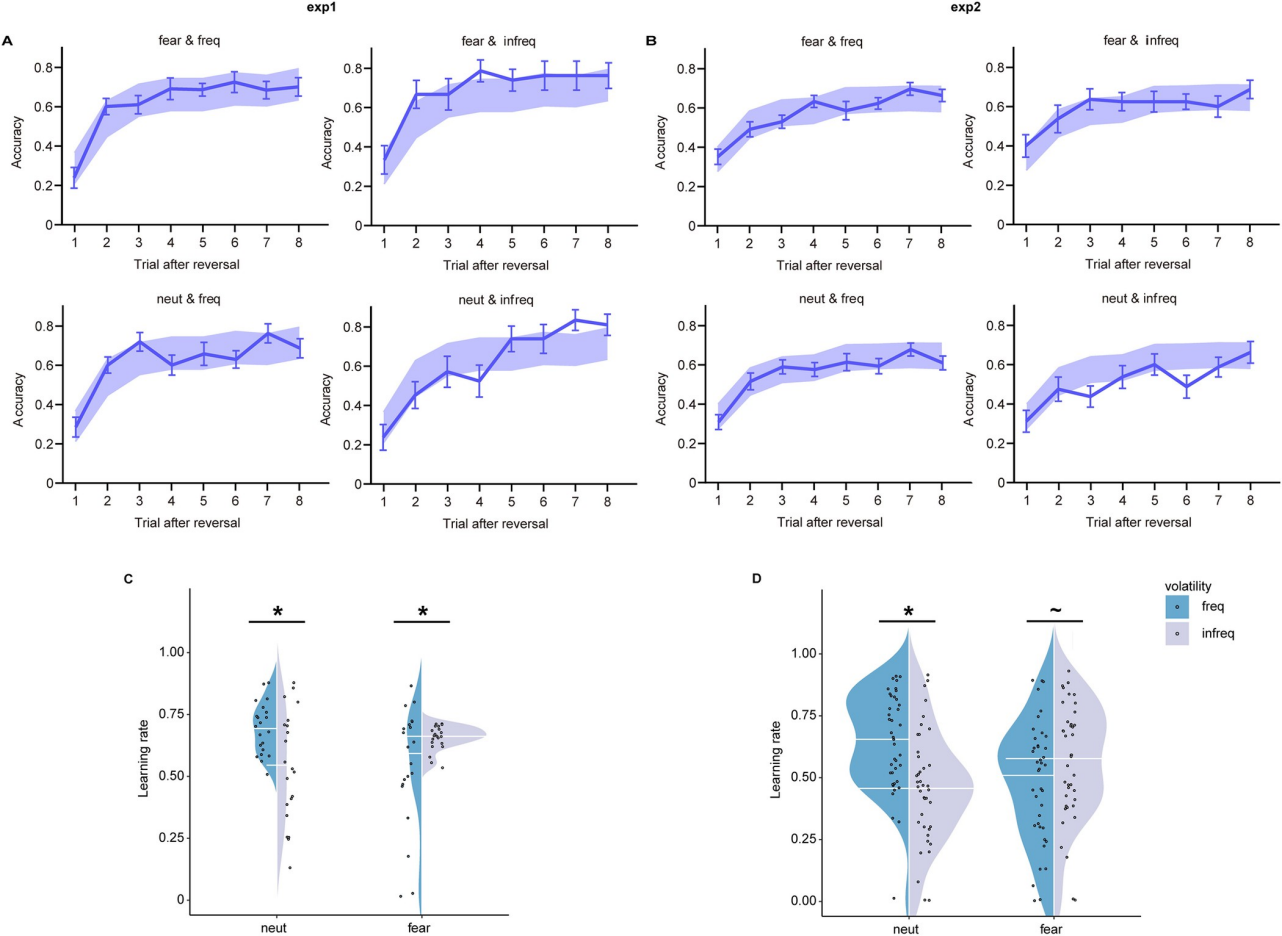
Behavioral results. (A, B) Performance after reversals in each condition of 2 experiments. Solid lines represent real data (mean ± standard error). Shaded panels represent 95% HDI from the winning model. (C, D) Learning rate from the winning model. The line within the violin plot represents the median value. Note: *p < 0.05; ^~^*p* < 0.1. The data underlying this figure can be found at https://osf.io/avhne/. HDI, high density interval.

We first tested the validity of fear induction (i.e., presentation of fearful/neutral facial expressions before each trial). We collected rating data for fearful and neutral facial expressions of “how fearful do you feel when seeing this face” using a Likert scale from 0 (no experience of fear at all) to 8 (strong experience of fear). Paired-sample *t* test showed a significant stronger fearful experience for fearful versus neutral expressions (*p* = 0.001; the mean rating for fearful expressions was 3.52; see S5 Text and S3 Fig), suggesting that participants indeed experienced slight-to-moderate fear during the experiment. To examine whether participants were able to learn the reward structure of our task, in line with Piray and colleagues (2019) [36], we plotted participants’ performance after reversal. Results showed that participants learned the reward schedule successfully (Fig 2A and 2B). In addition, a small number of missing trials (less than 14; <5.8%) and a low ratio of short response time (<200 ms; <1.67%; S2 Table) suggested sufficient engagement during the task.

Computational mechanisms underlying fear-biased adaptation to volatility learning

To formally quantify cognitive mechanisms underlying fear-biased adaptation to volatility learning, we constructed 12 models, with considerations of learning by trial and error (Rescorla–Wagner (RW) model), attentional lapses, forgetting, and learning by attention (Pearce-Hall model; see Materials and methods). Using indices of leave-one-out information criterion (LOOIC) and widely applicable information criterion (WAIC), model comparison showed that the winning model was Model 1 (M1) in both exp1 and exp2 [except slightly better for model 5 (M5) at the index of LOOIC in exp2, ΔLOOIC = −0.4; Table 2]. Specifically, M1 assumed that each condition was learned differently with the trial-and-error strategy (1 learning rate per condition). The pseudoR2 and balanced accuracy for M1 were 0.247% and 64.9% for exp1 and 0.189% and 61.1% for exp2, which were decent [37]. The balanced accuracy in both exp1 and exp2 was significantly better than the chance level (i.e., 50%; *ps* < 0.001). We further validated this winning model (M1) in 4 ways for both exp1 and exp2. First, model simulation from M1 showed high correlation coefficients between real accuracy and simulated accuracy for every condition (exp1: *rs* > 0.82, *ps* < 0.001; exp2: *rs* > 0.85, *ps* < 0.001; S4 Fig). Second, model simulation from M1 showed high correlations between real and simulated win-stay and loss-switch (WSLS) behavior for each condition (exp1: *rs* > 0.80, *ps* < 0.001; exp2: *rs* > 0.78, *ps* < 0.001; S5 and S7 Figs). Third, parameter recovery for M1 showed high correlations between real parameters and simulated parameters (see S2 Text and S6 Fig). Finally, model recovery for M1 showed that the winning model among 12 candidate models was identifiable (see S2 Text and S3 Table). In addition, simulated data closely resembled the real performance (Fig 2A and 2B), confirming the validity of parameter estimation.

**Table 2:** Model comparison.

Models	Number of parameters	exp 1 (*n* = 21)				exp 2 (*n* = 40)			
		ΔLOOIC	ΔWAIC	pseudoR2	BA (%)	ΔLOOIC	ΔWAIC	pseudoR2	BA (%)
M1	8	0	0	0.247	64.9	0	0	0.189	61.1
M2	4	11.3	20.7	0.237	64.5	103.4	118.5	0.172	60.3
M3	5	7.6	23.0	0.238	64.6	35.9	53.2	0.180	60.7
M4	8	64.9	68.2	0.234	63.8	112.2	126.8	0.179	60.4
M5	9	24.6	57.8	0.243	64.8	−0.4	11.5	0.187	61.1
M6	9	118.7	89.7	0.224	63.9	107.6	112.5	0.179	60.7
M7	10	74.5	85.1	0.233	64.3	136.4	135.2	0.178	60.7
M8	10	62.0	70.4	0.234	64.3	123.7	128.1	0.178	60.6
M9	7	53.6	63.0	0.233	63.7	124.9	131.6	0.175	60.1
M10	8	53.9	59.0	0.233	63.7	123.9	131.0	0.175	60.1
M11	8	51.7	58.1	0.233	63.7	112.8	124.4	0.176	60.2
M12	11	2.6	8.9	0.246	64.9	35.3	57.5	0.187	61.0

The winning model in both exp1 and exp2 is M1.

ΔLOOIC, leave-one-out information criterion relative to the winning model; ΔWAIC, widely applicable information criterion relative to the winning model; BA, balanced accuracy.

To examine how learning rates from the winning model M1 were modulated by emotional cue and environmental volatility, we implemented linear mixed-effect models (LMMs) with subject as a random factor and with cue (fear/neut) and volatility (freq/infreq) as within-subject factors for exp1 and exp2, respectively. We observed a significant interaction effect between cue and environmental volatility in each experiment (exp1: F = 19.095, *p* < 0.001, partial *η*^2^ = 0.24; exp2: F = 22.509, *p* < 0.001, partial *η*^2^ = 0.16; Fig 2 and 2D). Simple effect analysis consistently showed a higher learning rate for environments with freq versus infreq in neutral cues (exp1: F = 13.126, *p* = 0.004, partial *η*^2^ = 0.18; exp2: F = 22.667, *p* < 0.001, partial *η*^2^ = 0.16), replicating previous results regarding adaptation to volatility learning [14,17,24]. However, this pattern disappeared or reversed in the face of fearful cues (exp1: F = 6.538, *p* = 0.079, partial *η*^2^ = 0.10, freq < infreq; exp2: F = 3.799, *p* = 0.322, partial *η*^2^ = 0.03). These findings suggest that fear interferes with adaptation to volatility learning. To identify whether the manipulated variables (volatility and cue) influence learning rates, rather than inverse temperature, we also added inverse temperature as the covariate in the regression model. Results showed the same pattern (see S1 Text for details), suggesting that the manipulated variables specifically impacted learning rates. In addition, M5, adding a component of attentional lapse, performed slightly better than M1 at the index of LOOIC in exp2 (ΔLOOIC = −0.4; Table 2). The implementation of LMM for M5 showed the same pattern as M1 (S9 Fig), suggesting that the current results were highly robust. We also found a higher proportion to select the optimal option in the third trial after reversal for environments with frequent versus infrequent reversals in face of neutral cues (F = 9.622, *p* = 0.003, partial *η*^2^ = 0.14), while a reversal pattern was observed when cued by fearful facial expressions (F = 4.291, *p* = 0.043, partial *η*^2^ = 0.07), showing the same pattern with conditional differences in learning rates. It suggests that atypical mental computations for adaptation learning under fear result in aberrant learning performance, especially after reversal (see S2 Text and S8 Fig). Details of statistical results were reported in S1 Text and S2 Text.

Furthermore, to test the influences of fear on punishment learning, we conducted another experiment with the same design [2 (fear/neut) by 2 (freq/infreq)] under the punishment context (expS1 in S4 Text). We observed the same pattern with our findings in the reward context, suggesting that fear-biased adaptation to volatility is reward/punishment-independent (for details, see S4 Text and S10 Fig). We also conducted another experiment (expS2 in S4 Text) with the design of 2 (happy versus neutral facial expressions) by 2 (freq/infreq) in the reward context to control for the potential impact of attention-grabbing (without experiencing fear) on adaptation to volatility. Happy expressions were used here because fearful and happy expressions have similar properties of attention-grabbing but distinct affective experience [38]. We did not find a significant interaction effect between cue and volatility (S11 Fig), suggesting that fear experience, but not the attentional-distracting property, disrupts flexible adaptation to volatility (see S4 Text for details).

Neural correlates of fear-biased adaptation to volatility learning: Learning rates

**Fig 3:**
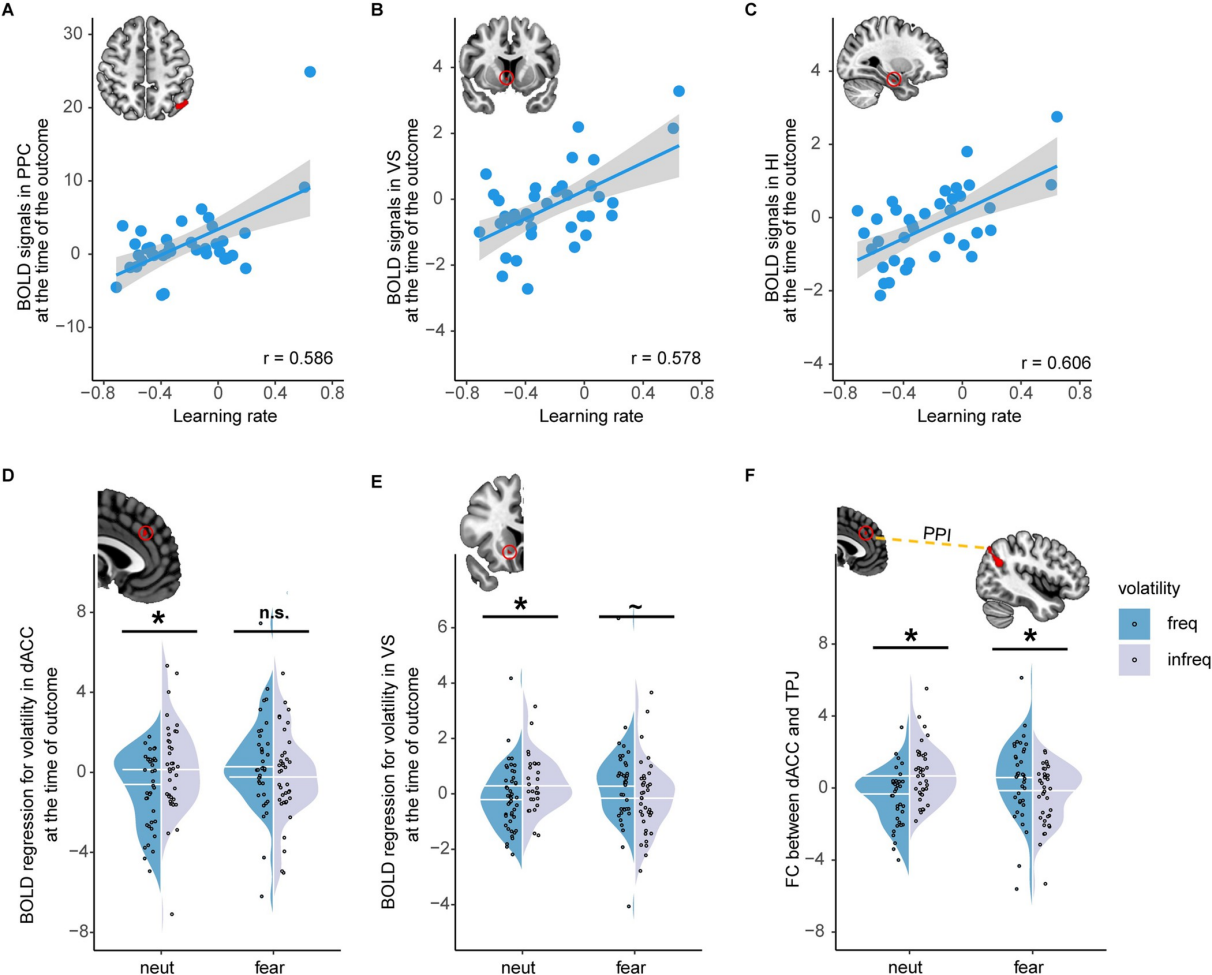
Activity and connectivity results. Neural correlates of fear-biased adaptation to volatility learning in terms of learning rate in the (A) PPC (whole-brain analysis, peak at [–50 –60 50], k = 103), (B) VS (ROI analysis, peak at [–4 8 –4], k = 7), and (C) HI (ROI analysis, peak at [28 –16 –22], k = 10). Activation results from fear-biased adaptation to volatility learning in terms of subjective volatility in the (D) dACC (ROI analysis, peak at [–2 32 32], k = 8) and (E) VS (ROI analysis, peak at [10 16 –8], k = 9). (F) FC between the dACC (seed region) and TPJ (target region, whole-brain analysis, peak at [–64 –52 36], k = 263) for each condition. Significant voxels are computed across the 2 conditions. Circles in red represent ROI analysis. Activation maps without the red circle represent whole-brain analysis. Both the scatter and violin plot show the mean activation within the corresponding cluster. The line within the violin plot represents the median value. Note: PPC, posterior parietal cortex; VS, ventral striatum; HI, hippocampus; dACC, dorsal anterior cingulate cortex; FC, functional connectivity; n.s., not significant; *p < 0.05; ^~^*p* < 0.1. The source data can be found at https://osf.io/avhne/.

To examine how fear-biased adaptation to volatility learning was represented in the human brain from the perspective of learning rates, we performed the first generalized linear model (GLM1). The behavioral bias was defined as [(fear & freq–fear & infreq)–(neut & freq–neut & infreq)] in learning rates, whereas the neural bias was defined as [(fear & freq–fear & infreq)–(neut & freq–neut & infreq)] in BOLD signals at the outcome stage. This study focused on the dACC, VS, amygdala, OFC, and HI that have been identified to link emotion to adaptation to environmental volatility (see Introduction for details). The dACC was selected from Behrens and colleagues [14] (MNI: x = −6, y = 26, z = 34 mm, a sphere with 10 mm radius), a classical study on adaptation to volatility with fMRI. To keep the selective procedure objective, other ROIs were obtained from AAL atlas. Small volume correction (SVC) was used for ROIs. All imaging results were corrected with the threshold of *p* < 0.001 at the voxel level and with the threshold of *p* < 0.05 at the cluster level using AlphaSim procedure (implemented in DPABI toolbox: version 3.1 [39]). These brain regions were thus hypothesized to contribute to fear-biased adaptation learning. Note that the coordinates for activated brain areas we reported represent peak coordinates, which were from second-level contrasts of the group. ROI analysis showed positive correlations between the behavioral bias and the neural bias in the VS (ROI analysis, peak at [4 8 –4], r = 0.578, *p* < 0.001, k = 7; Fig 3B) and HI (ROI analysis; peak at [28 –16 –22]; r = 0.606, *p* < 0.001, k = 10; Fig 3C). No significant activation in the amygdala, dACC, and OFC was found. We also conducted exploratory whole-brain analysis, revealing a positive correlation between the behavioral bias and the neural bias in the posterior parietal cortex (PPC; whole-brain analysis; peak at [–50 –60 50] in MNI coordinates; r = 0.586, *p* < 0.001, k = 103; Fig 3A). These findings remained significant when controlling for potential differences in outcome (see S3 Text for details). These results suggest that the PPC, VS, and HI encode fear-biased adaptation to volatility learning.

Neural substrates of fear-biased adaptation to volatility learning: Subjective volatility

To examine representations of the human brain for fear-biased adaptation to volatility learning, we implemented model-based fMRI analysis (GLM2) with parametric modulation of subjective volatility at the stage of outcome. Again, the neural bias was defined as [(fear & freq–fear & infreq)–(neut & freq–neut & infreq)] in the parametric activation of subjective volatility. Note that trial-by-trial subjective volatility was derived from Bayesian Learner model (see Materials and methods for detailed equations; see S12 Fig for an example participant). We observed significant activations in the dACC (ROI analysis; peak at [–2 32 32]; k = 8, Fig 3D) and VS (ROI analysis; peak at [10 16 –8]; k = 9; Fig 3E). We also validated significant activation in the dACC with different sphere radiuses (e.g., 8 mm and 12 mm; see S3 Text for details). No significant activation in the amygdala, HI, or OFC was found. Specifically, for both the dACC and VS, we first observed significant interaction effects between cue and environmental volatility (dACC: F = 14.609, *p* < 0.001, partial *η* [2] = 0.29; VS: F = 15.274, *p* < 0.001, partial *η* [2] = 0.30). Simple effect analysis showed significant increased activation in infreq versus freq with neutral cues (dACC: F = 13.272, *p* = 0.001, partial *η*^2^ = 0.27; Fig 3D; VS: F = 8.191, *p* = 0.007, partial *η*^2^ = 0.19; Fig 3E). However, such patterns disappeared or reversed for fearful cues (dACC: F = 1.833, *p* = 0.184, partial *η*^2^ = 0.05; Fig 3D; VS: F = 3.966, *p* = 0.054, partial *η*^2^ = 0.10; Fig 3E). These findings remained significant when controlling for potential differences in outcome (see S3 Text for details). These results indicate that the dACC and VS are engaged to encode subjective volatility in fear-biased adaptation.

We next sought to explore the underlying brain networks modulating fear-biased adaptation to volatility learning. Based on model-based fMRI results, generalized psychophysiological interaction (gPPI) [40] analysis was performed with the dACC and VS as seed regions, separately. We found significant connectivity of the dACC with the temporal parietal junction (TPJ; whole-brain analysis; peak at [–64 –52 36]; k = 263; Fig 3F). The interaction effect (F = 26.551, *p* < 0.001, partial *η* [2] = 0.42) further showed that functional connectivity between the dACC and TPJ increased in infreq as compared to freq following neutral cues (F = 11.306, *p* = 0.002, partial *η*^2^ = 0.24), but decreased following fearful cues (F = 4.748, *p* = 0.036, partial *η*^2^ = 0.12). The gPPI results suggest that the dACC-TPJ circuit encodes subjective volatility calculations in fear-biased adaptation.

**Fig 4:**
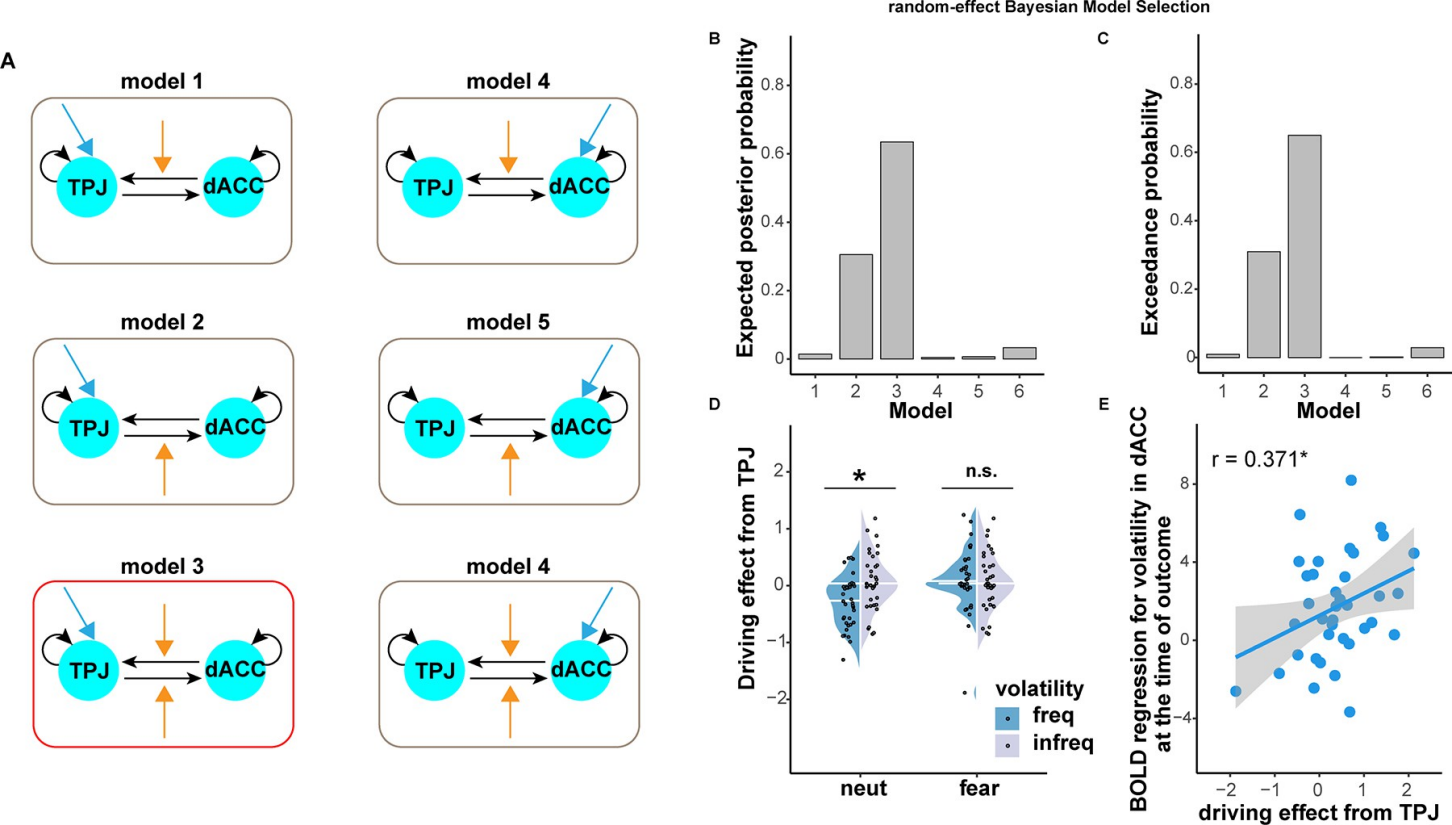
DCM results. (A) Model space for DCM analysis. Black arrows represent intrinsic connectivity. Blue arrows represent the driving effect. Orange arrows represent modulatory effects. The winning model was highlighted in red. Model comparison using indices of (B) expected posterior probability and (C) exceedance probability. (D) Driving effect on TPJ for each condition. The line within the violin plot represents the median value. (E) Correlation between driving effect on the TPJ and parametric activation of subjectivity volatility in the dACC in terms of fear-biased adaptation to volatility learning. The scatter plot shows the mean activation within the corresponding cluster. Note: TPJ, temporal parietal junction; dACC, dorsal anterior cingulate cortex; RFX, random-effect; n.s., not significant; *p < 0.05. The source data can be found at https://osf.io/avhne/.

To further test directions of information flow between the dACC and TPJ underlying fear-biased adaptation to volatility, we conducted dynamic causal modeling (DCM) analysis [41]. We constructed 6 models with different assumptions of modulatory effects and driving effects while we fixed the full intrinsic connectivity (Fig 4A; see Materials and methods for details). The random-effect (RFX) Bayesian model selection (BMS) using indices of exceedance probability and expected posterior probability recommended model 3 as the winning model (Fig 4B and 4C). The model 3 assumed a driving effect from the TPJ, a modulatory effect from the TPJ to dACC, and another modulatory effect from the dACC to TPJ. We found a significant interaction effect between cue and environmental volatility in the driving effect on the TPJ (F = 8.321, *p* = 0.007, partial *η*^2^ = 0.19; Fig 4D). Simple effect analysis showed a significant increase in infreq than freq in neutral cues (F = 9.827, *p* = 0.003, partial *η*^2^ = 0.21; Fig 4D), but not fearful cues (F = 0.064, partial *η*^2^ = 0.00; Fig 4D), suggesting the modulation of the driving effect from the TPJ on fear-biased adaptation to subjective volatility. Given that the winning model (DCM model 3) was close to model 2, as shown by exceedance probability for model 3 (about 0.6) and model 2 (about 0.3; Fig 4C), we also checked differences in the driving effect on the TPJ. Results showed the same pattern with the winning model (S1 Text), indicating the robustness of this finding. Furthermore, the driving bias from the TPJ was positively correlated to parametric bias for BOLD signals in subjective volatility of the dACC (r = 0.371, *p* = 0.024; Fig 4E). Taken together, consistent with our behavioral modeling results, these neural results relating to subjective volatility support the notion that fear interferes with adaptation to volatility learning and further uncover the driven computation from the TPJ in the dACC-TPJ pathway underlying fear-biased adaptation to volatility learning.

### Alexithymia was related to fear-biased adaptation to volatility learning

**Fig 5:**
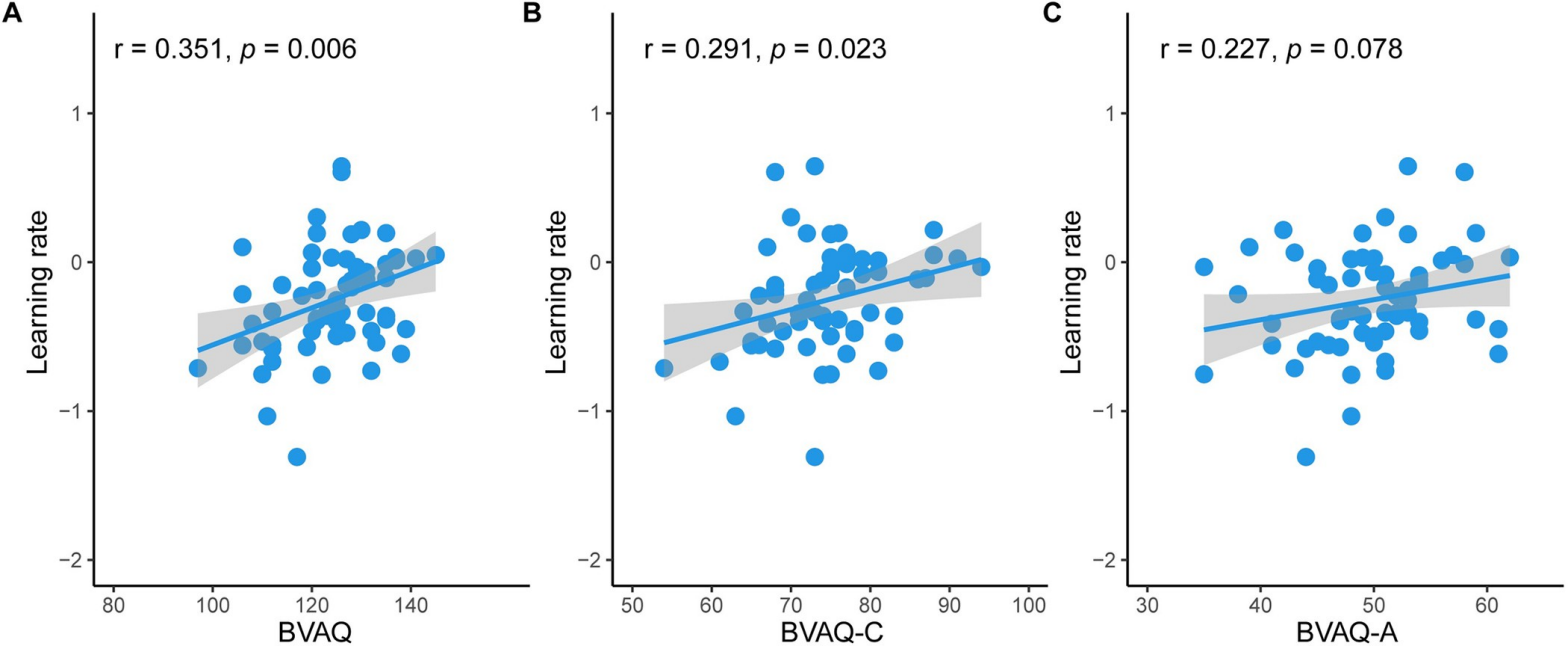
Correlations of learning rate in terms of fear-biased adaptation to volatility learning with BVAQ (A), BVAQ-C (B), and BVAQ-A (C). Note: BVAQ, the Bermond–Vorst Alexithymia Questionnaire; BVAQ-C, cognitive dimension of BVAQ; BVAQ-A, affective dimension of BVAQ. The source data can be found at https://osf.io/avhne/.

We found a significant correlation between the total score of the Bermond–Vorst Alexithymia Questionnaire (BVAQ) [42] and the behavioral bias (in learning rates; r = 0.351, *p* = 0.006; Fig 5A). Please note that higher scores on the BVAQ represented lower levels of alexithymia. The higher score for behavioral bias [(fear & freq–fear & infreq)–(neut & freq–neut & infreq)] reflected weaker influence of fear on adaptation to volatility learning. Therefore, the positive correlation suggests that the interference of fear with adaptation to volatility learning was stronger the more alexithymic individuals were. To examine contributions of cognitive (BVAQ-C) and affective dimensions (BVAQ-A) of alexithymia for learning rate bias, we performed correlations of learning rate bias with BVAQ-C and BVAQ-A, respectively. We observed a significant correlation of learning rates-bias with BVAQ-C (r = 0.291, *p* = 0.023; Benjamini–Hochberg FDR corrected; Fig 5B), while the correlation with BVAQ-aff was not significant (r = 0.227, *p* = 0.078; Fig 5C), indicating that the cognitive dimension of alexithymia explained fear-biased adaptation to volatility learning. In addition, these results hold when controlling for anxiety and depression (see S2 Text).

## Discussion

This study examined neuro-computational mechanisms of how fear influenced adaptation to volatility. In 2 independent experiments, we consistently found that the environment with frequent reversals elicited a higher learning rate than the environment with infrequent reversals in neutral cues, replicating previous studies [14,17,24]. However, this difference was absent in the face of fearful cues, supporting the hypothesis that fear prevented adaptation to volatility. This suppressive effect was underpinned by activity of the PPC, VS, HI, and dACC, as well as functional connectivity between the dACC and temporal-parietal junction (TPJ), suggesting distributed brain systems for computations of fear-biased adaptation. Effective connectivity results further showed that this bias mainly resulted from the driving effect from the TPJ, indicating that computations underlying fear-biased adaptation to volatility may occur at a relatively early stage of processing bottom-up inputs. Lastly, this bias was stronger in individuals with higher levels of alexithymia.

Using computational modeling, this study revealed that fear inhibits adaptation to volatility. In our control conditions of neutral cues, participants showed a higher learning rate to the environment with frequent (as compared to infrequent) reversals, consistent with previous findings of a higher learning rate for volatile than stable conditions [14,17,24]. This finding supports the notion that humans are able to adjust learning rates in adaptation to volatility [14,16,17,24]. Critically, this adjustment was disrupted by fearful signals. It has been shown that fear disrupts systems supporting flexible behavior through dominating consciousness [6,10,11]. Therefore, the conscious experience of fear potentially inhibits adaptive function, in particular adaption to volatility. This supports the notion of mutual inhibition between emotion and cognition and the dual competition model of emotion–cognition integration [30,43,44]. In addition, we observed that fear prevented flexible adjustments to volatility in both reward and punishment contexts (see S4 Text), suggesting that our findings of fear-biased adaptation to volatility were reward/punishment-independent. It seems that our manipulation was similar to those from Browning and colleagues (2015) and observed inconsistent results. Although electrical shocks used in Browning and colleagues (2015) also evoked strong fear, our study systematically examined the role of fear (as compared to the neutral baseline) on adaptation to volatility. Importantly, their manipulation of fear was contingent on the outcome (shock or not shock), whereas fearful facial expressions in our study were associated with environmental volatility, which is a higher level in the Bayesian Learner model [14]. Previous studies have shown different representations between outcome encoding and volatility processing [45,46]. It has also been shown inflexible adaptation to volatility in anxious individuals [17]. Given the high similarity in concepts and structures between anxiety and fear [47], we complemented Browning and colleagues (2015)’s findings from individual difference in anxiety to the within-subject effects of fearful cues. Together, these findings jointly suggest that fear-biased adaption to volatility characterizes fear-related affective disorders.

Neuroimaging results showed that the dACC encoded fear-biased adaptation to volatility learning. In our control conditions, decreased activation in the dACC was observed in environments with frequent versus infrequent reversals. This finding was contrary to previous findings of a positive correlation of dACC signals with subjective volatility [14,26]. However, it has been proposed that task difficulty modulates the relationship between activation in the dACC and cognitive demand in an inverted U-shape pattern [48,49]. For example, stronger dACC activation has been found for processing fearful versus neutral faces under low cognitive demand (e.g., 0-back task), while lower responses of the dACC to fearful versus neutral expressions have been observed under high cognitive demand (e.g., 2-back task) [30]. One explanation for the discrepancy in activation of the dACC might be that the current task was more difficult than those of the previous probabilistic reward learning task due to our design of interleaved trials from 4 conditions. Importantly, the signal of volatility in the dACC was absent in fearful conditions, suggesting a suppressive role of fear in the adaptive function of the dACC. The dACC is considered to be a higher-order integrative hub for multiple information [30,50,51]. For example, integration between fearful signals and executive functions has been found in the dACC [30]. Therefore, these results indicate that fear modulates the dACC’s signals to track environmental volatility and support the integrative processing of emotion and cognition in the dACC.

The TPJ was also found to work in concert with the dACC to influence fear-biased adaptation to volatility in our study. A nexus model of the TPJ has contended a basic integrative hub for the TPJ function, including attentional processing, memory storage, and social processing [52]. For example, the TPJ was identified to be involved in global Gestalt integration at the perceptive level [53]. Moderation of dACC-TPJ functional connectivity on fear-biased adaptation to volatility in our study suggests a circuit of information interchange between low- and high-level integrative processing of fear and adaptation learning. While bidirectional information flow between the dACC and TPJ during fear-biased adaptation learning was observed, fear-biased adaptation to volatility was influenced by the driving effect from the TPJ. This may suggest that fear mainly impedes bottom-up information input and low-level integrative processing between fear and adaptation to volatility. In sum, in combination with the significant correlation between the activated bias in the dACC and the driving bias in the TPJ (Fig 5E), these results reveal a crucial role of the TPJ-dACC neural pathway in volatility learning of fear-biased adaptation.

We also observed activation in the VS, but not the amygdala, in fear-biased adaptation to volatility, which suggests differential roles of the VS and amygdala. Both the VS and amygdala have been demonstrated to represent uncertainties during dynamic environments and emotion processing [27,54–56]. Within learning contexts, the amygdala is linked to Pavlovian conditioning, while the VS has been implicated in instrumental learning [6]. The current results may thus reflect the interference of instrumental adaptation by fear in the VS. Another explanation regards a possible role of Pavlovian-instrumental transfer. This transfer effect underlying the amygdala and VS has been demonstrated in animal models [6,57]. In this study, Pavlovian tendencies were gradually dominated by instrumental learning. This transfer effect thus explains activation in the VS, but not the amygdala, in fear-biased adaptation learning. However, interpretations about the negative result of activation in the amygdala should be cautious. In addition, the HI and PPC were found to moderate fear-biased adaptation to volatility in our study. The HI has been thought to encode and store memory [58,59], while the PPC was implicated in memory retrieval [60]. These 2 brain regions were also involved in learning and value representation in the uncertain environment [13,28,61,62]. Therefore, these results indicate that fear interferes with memory signals in the HI and PPC for adaptation to dynamic environments. Our study did not find any significant OFC activation. This is surprising given its crucial involvements in emotion-related valuation and flexible learning [29,63,64]. One potential explanation is low signal-noise ratio for the OFC BOLD signals [65]. In brief, distributed brain areas involved in memory and learning systems seem to contribute to fear-biased adaptation to volatility learning.

This study used the conventional reinforcement learning model and the Bayesian Learner model, which are complementary to each other regarding cognitive and neural mechanisms and behaviors [66]. We found shared (e.g., activation in the VS) and distinct neural mechanisms between these types of models. Unexpectedly, no direct correlation between learning rate and subjective volatility was observed in this study. The Bayesian Learner model has been proposed in a hierarchical structure [14] with (i) subjective estimation of environmental volatility at the first level; (ii) subjective estimation of the winning probability at the second level; and (iii) the observed outcome at the third level. Based on the reinforcement learning model without hierarchical processing, learning rates were fit from the subjective probability across trials at the second level. Previous studies indeed showed a positive correlation between volatility signals and learning rate [14,17]. However, a recent simulation study demonstrated that learning rates were jointly estimated from both stochasticity and volatility [16]. Although separate correlations of activation in the HI were observed with the volatility index and the learning rate, the absence of a direct correlation between volatility and learning rate may be due to the ignorance of stochasticity in our model space, which was in line with previous studies [14,17,24]. Future studies may benefit from taking stochasticity into account when constructing computational models to examine the relationship between learning rate and volatility.

We observed that maladaptation to volatility following fear cues correlated with alexithymia, suggesting a stronger influence of fear on adaptation to volatility learning in individuals with higher levels of alexithymia. Alexithymia refers to difficulties in emotion processing [34]. Alexithymia has been considered as a transdiagnostic risk factor for various affective disorders, such as anxiety and depression [67,68]. While a rich literature showed both cognitive and emotional deficits in alexithymia [35,49,69,70], the current results further suggest an integrative difficulty between fear and adaptation to volatility in individuals with high alexithymia levels. Given that abnormalities in regulating fearful signals are at the core of affective disorders [6] and failure to appropriately adjust learning to dynamic environments contributes to affective disorders [17,22–24], our findings provide additional insights into pathological mechanisms of alexithymia-related affective disorders.

Several limitations of the present study should be mentioned. First, in addition to fear-biased adaptation to volatility in terms of learning rates, we also observed that fearful relative to neutral faces induced hypo-learning speed during infrequent reversal environments and hyper-learning rate for environments with frequent reversals, suggesting suboptimal learning under fear. Future studies would be benefit from investigating whether fear influences adaption to volatility (differences between frequent and infrequent reversal environments) or fear induces suboptimal learning in environments both with frequent and infrequent reversals, resulting in the observed maladaptation. Second, there are different variants for Bayesian Learner model, e.g., hierarchical Gaussian filter [21]. We followed the most classical research on adaptation to volatility and only used Bayesian Learner model [14]. Future studies would benefit from examining shared and distinct neural responses among these variants. Last, participants indeed reported fear in response to fearful expressions. Although subjective report has been considered to be importance in the measurement of fear [3,4], future studies would benefit from real-time objective measures using physiological recordings (e.g., electrocardiography and eye-tracking) to examine the interaction between emotion swing and learning during uncertainty.

To conclude, this study provides a neuro-cognitive account of how fear interferes with adaptation to volatility during dynamic environments at the computational level. We also show that this bias is related to individual differences in propensity for emotion processing and regulation. Our findings reveal distributed brain substrates underlying fear-biased adaptation to volatility, including integration systems, learning systems, and memory systems. The TPJ-dACC pathway, in particular, influences the interplay between fear and adaptation to volatility learning, suggesting that the suppression of fear on adaptive behaviors may occur at a relatively early stage of processing bottom-up inputs. Our work thus sheds light on the neuro-computational mechanisms underlying fear-biased adaptation to volatility learning.

## Materials and methods

### Participants

Sixty-four right-handed healthy college students participated in 2 experiments. Experiment 1 (exp1) was a behavioral study, including 21 participants (11 females, age = 20.81 ± 1.94). Experiment 2 (exp2) was an fMRI study, including 43 participants. Due to impatience (1 participant), and many missing trials (2 participants failed to respond in more than 10% of the trials; 62 and 46 in the 240 trials, respectively), and excessive head motion (3 participants with more than 10% scans with frame-wise displacement (FD) > 0.5) [71], the final sample for exp2 included 40 participants (18 females, age = 21.75 ± 2.13) for behavioral analysis and 37 participants (16 females, age = 21.57 ± 2.06) for fMRI analysis. See Table 1 for demographic information. All the participants had no history of neurological and psychiatric disorders or head injury. The study was approved by the Center for Brain Disorders and Cognitive Sciences Institutional Review Board (IRB number: CBDCS202107080020) at Shenzhen University and performed in full compliance with the latest Declaration of Helsinki. Written informed consent was obtained from all participants.

### General procedure

After signing the informed consent, participants were asked to complete a three-stage training task to understand the probability and reversal components underlying the task [72]. The cue-biased adaptation learning task was then implemented (while participants in exp2 simultaneously experienced fMRI scanning). After the task, participants were asked to complete the Chinese version of the BVAQ [42] and the Mood and Anxiety Symptoms Questionnaire (MASQ) [73]. Finally, participants were paid based on their learning performance, which was instructed before the experiment.

### Cue-biased adaptation learning task

Inspired by Behrens and colleagues (2007) [14] and Piray and colleagues (2019) [36], we developed a cue-biased adaptation learning task (Fig 1). Using the framework of the probabilistic reward reversal learning task [14,24], we manipulated the type of cue (fearful/neutral expressions) and environmental volatility (frequent/infrequent reversals) [16]. Specifically, frequent reversals (freq) were defined as a situation in which the contingency reversed every 9 to 11 trials randomly, while infrequent reversals (infreq) referred to a situation in which the contingency reversed every 18 to 22 trials randomly. In brief, the current study with a two by two within-subject design consisted of 240 trials, with 60 trials per condition. Please note that trials for each condition were intermixed within a block. Participants were asked to learn by trial and error to maximize their payoff. Although participants were explicitly informed that reward structure would change throughout the task, they needed to infer the moment on which reversal occurred and the speed at which reversal changed [45]. At the beginning of each trial, a cue (a fearful or neutral face) was first shown on the screen center with duration of 1 s (Fig 1A). To eliminate potential gender effects, facial expressions with the same gender with participants were used as cues. For example, the female face with the fearful or neutral expression was shown on the cue for the female participants. We selected 4 female faces and 4 male faces from the Taiwanese Facial Expression Image Database (TFEID) [74], with 2 fearful and neutral expressions for each gender. The normative rating (category and intensity) from the TFEID can be seen in S1 Table. Please note that the cue type and the environmental volatility were randomly matched across participants. After a fixation cross with a random duration (0.2 to 1.5 s for exp1 and 1 to 3 s for exp2), 2 options (horizon and vertical Gabor patches) were presented randomly on each side. Participants were required to make a decision within 2 s. Upon response, the selected option would be highlighted for 0.2 s, followed by a question mark at the screen center with a jitter interval at 1 to 3 s. Then, the outcome was shown for 1 s, with “+1” in green indicating reward and “+0” in red reflecting no reward. Outcome was delivered with a probability of reward at either 85% or 15%, based on the outcome schedule (Fig 1B). If participants did not respond within 2 s, which was defined as a missing trial, the option would not be highlighted and outcome was “+0.” At the end of a trial, a fixation cross was presented for 0.3 to 5.6 s to ensure that each trial lasted for 9 s for exp1 and 0.8 to 6.8 s to ensure that each trial lasted for 11 s for exp2. Exp1 and exp2 lasted for 36 and 44 min, respectively. All experimental procedures were presented using E-prime 2.0 (Psychology Software Tools, Pittsburgh, Pennsylvania, United States of America).

### Self-report questionnaires

To assess alexithymia, we used the Chinese version of the BVAQ [42]. This questionnaire consists of 35 items, each answer being scored on a five-point Likert scale of 1 (this in no way applies to me) to 5 (this definitely applies to me). BVAQ includes both cognitive and affective components with acceptable reliability and validity. Higher scores for BVAQ represented lower levels of alexithymia. To control for potential confounding effects of anxiety and depression, participants also completed the Chinese version of the MASQ [73]. The MASQ consists of 62 items that are assessed with a four-point Likert scale of 1 (not at all) to 4 (extremely). It measures symptoms of anxiety and depression based on a tripartite model, including general distress, which can be further divided into general distress: anxiety (GDA) and general distress: depression (GDD), anxious arousal (AA), and anhedonia depression (AD) subscales.

### Computational modeling of task performance

To best describe participants’ learning performance in our fear-biased volatility learning task, we conducted a stage-wise model construction procedure [24,75]. That is, we added each component to the model or modified an existing component progressively, based on the best model from the previous stage. Model comparison used the LOOIC and the WAIC to avoid overfitting. Lower scores of LOOIC or WAIC indicated better out-of-sample prediction accuracy of the candidate model. Parameter estimation was performed using hierarchical Bayesian analysis. Posterior inference was performed with Markov chain Monte Carlo (MCMC) sampling with 4,000 iterations across 4 chains from the posterior distribution. The entire modeling-related procedures were performed using the hBayesDM package [76]. In total, we tested 12 candidate models using the stage-by-stage model construction procedure in exp1. For exp2, we compared these models directly.

We used the simple RW model (Eqs 1–3) as the baseline model [18], which was widely used in learning-related studies [14,17,24]. The simple RW model assumed that participants learned reward structure by trial and error (Eqs 1–3) [18].


$$V_{c,t}=V_{c,t-1}+\alpha (O_{t-1}-V_{c,t-1})$$ (1)

$$V_{nc,t}={1-V}_{c,t}$$ (2)

$$P(A)=\frac{1}{1+e^{-\beta (V_{A}-V_{B})}}$$ (3)

Here, the value V of the chosen option is updated trial-by-trial, which are determined by both the prediction error and learning rate *α* (0 < *α* < 1). The prediction error is derived from the difference between received outcome (O) and expected value (V) from the previous trial. For simplicity, the value of the unchosen option is regarded as the opposite value for the chosen option [14,17]. Finally, we used a softmax function with a decision parameter *β* (or inverse temperature parameter; 0 < *β* < 10) to calculate the chosen probability for each option.

In Stage 1, we compared a model (M1) that assumed each of 4 conditions was learned differently with a model (M2) that assumed that participants learned differently between volatility [14], but not the type of cue. That is, M2 assumed that there was no cue (emotional) effect for all parameters. Thus, M1 included a learning parameter (learning rate) and a decision parameter (inverse temperature) for each condition, whereas M2 consisted of 2 learning parameters and decision parameters for conditions of frequent and infrequent reversals. Given that it was well established that the environment with frequent reversals elicited higher learning rate than that with infrequent reversals [14,17,24], we thus did not include the model assuming that there was no effect of volatility. M1 with 8 parameters showed better performance than M2 with 4 parameters (Table 2).

In Stage 2, we removed/added some components to the M1 to validate the winning model from Stage 1. M3 assumed that participants learned differently for each condition but shared a decision parameter across conditions (5 parameters). M4 assumed that participants regarded our task as the two-arm bandit and only updated the value of the chosen option (8 parameters), rather than the one-arm bandit (Eq 2). M5 added a parameter of the lapse in attention (*ε*; 0 < *ε* < 1; Eq 4), assuming that participants occasionally made random choices due to a lapse in attention (9 parameters).


$$P(A)=(1-\varepsilon )\frac{1}{1+e^{-\beta (V_{A}-V_{B})}}+\frac{\varepsilon }{2}$$ (4)

Model comparisons among M1, M3, M4, and M5 indicated that M1 performed better (see Table 2).

Participants needed to learn 4 types of cue-option-outcome contingencies in our task (as compared to 1 type in the previous probabilistic reward reversal learning task), including the option of forgetting [77]. Therefore, in Stage 3, we added forgetting parameters *ϕ* to the M1 (0 < *ϕ* < 1; Eq 5). This parameter pulled the estimated value toward the random level (0.5).


$$V_{t}=V_{t-1}+\phi (0.5-V_{t-1})$$ (5)

M6 assumed a sharing forgetting parameter across conditions (9 parameters). M7 assumed *ϕ* were modulated by volatility, with 2 forgetting parameters for environments with frequent and infrequent reversals (10 parameters). Rather, we assumed *ϕ* were modulated by emotional cues in M8 (10 parameters). Model comparisons among M1, M6, M7, and M8 showed that M1 performed best (Table 2).

In Stage 4, we considered hybrid models of the Pearce-Hall model with the RW model given that this type of hybrid model performed best among the candidate models during the emotion- and volatility-related task [36]. M9 assumed a shared weighting parameter *ω* across conditions (0 < *ω* < 1; 7 parameters). M10 assumed distinct weighting parameters *ω* for fearful and neutral cues (8 parameters). M11 assumed different scale parameters *κ* of learning rate for fearful and neutral conditions (0 < *κ* < 1; 8 parameters). M9–11 were the same as M2, M4, and M5 in Piray and colleagues (2019), respectively. Again, M1 won among these candidate models (see Table 2).

We also added a model (M12) assuming linear relationships among 4 learning rates. Specifically, each parameter was linearly represented by the learning rate for the neutral and infrequent condition. M1 outperformed than M12 (Table 2), suggesting that it is better to independently represent each parameter in the current experiment, though there are significant correlations between each parameter.


$$\alpha_{\left(neut,freq\right)}=k_{\left(neut,freq\right)}\alpha_{\left(neut,infreq\right)}+b_{\left(neut,freq\right)}$$ (6)

$$\alpha_{\left(fear,infreq\right)}=k_{\left(fear,infreq\right)}\alpha_{\left(neut,infreq\right)}+b_{\left(fear,infreq\right)}$$ (7)

$$\alpha_{\left(fear,freq\right)}=k_{\left(fear,freq\right)}\alpha_{\left(neut,infreq\right)}+b_{\left(fear,freq\right)}$$ (8)

We further performed model validation for the winning model (M1) in exp1 using the generated data from MCMC sampling (4,000 iterations). First, we computed correlations between real accuracy and simulated accuracy for each condition. Please note that mean simulated accuracy across 4,000 iterations per condition and participant was used. Second, we analyzed the generated data using the index of performance after reversal and computed 95% high density posterior interval (HDI) to compare the difference between simulated data and real data.

Next, we compared these 12 candidate models directly in exp2. The same procedures of model validation were performed for exp2 behavioral data.

### Statistical analysis for learning rates

Each parameter was represented by the mean of the posterior distribution of the parameters. We conducted LMMs on learning rate with subject as a random factor and with cue (fear/neut) and volatility (infreq/freq) as within-subject factors for exp1 and exp2, respectively. Pearson correlations of fear-biased adaptation to volatility learning in terms of learning rate were implemented with total BVAQ scores, the cognitive dimension, and the affective dimension of BVAQ scores across 61 participants. Statistical analyses were conducted using SPSS 17.0 (IBM) and R (4.1.0). We set the significance level at *p* = 0.05.

### Bayesian learner model

Bayesian Learner model has been shown to dynamically track environmental volatility [14]. Specifically, the learner estimates the probability to gain reward on the next trial (*V*_*t*+1_) given the obtained outcome and the estimated probability on the current trial (*V*_*t*_). Please note that *V*_*t*_ can also be represented as probability given that the magnitude in our study was fixed at 1. Taking advantage of the Markovian assumption, the new estimated probability only depends on information from the last trial, but not the full history of previous trials. Therefore, the estimation of probability on trial t+1 can be represented using a beta distribution with the estimated probability on trial t as the mean and a width parameter sv, where sv equals to exp (SV), representing the estimated subjective volatility of the environment.


$$P(V_{t+1}|V_{t},SV)∼\beta (V_{t},sv)$$ (9)

Another parameter is a distrust parameter k, representing uncertainty in the current estimation of environmental volatility. Subjective volatility of the next trial (SV_t+1_) can be represented as a normal distribution, with *SV*_*t*_ as the mean and K as the width parameter, where K = exp(k).


$$P({SV}_{t+1}|SV_{t},k)∼N({SV}_{t},K)$$ (10)

The joint probability of V_t+1_, SV_t+1_, and k is estimated based on the outcome (y) of each trial:

$$P(V_{t+1},{SV}_{t+1},k|y_{\leq t+1})\propto P(y_{t+1}|V_{t+1})\int \left[\int P(V_{t},{SV}_{t},k|y_{\leq t})P({SV}_{t+1}|{SV}_{t},k)d{SV}_{t}\right]P(V_{t+1}|V_{t},{SV}_{t+1})dV_{t}.$$ (11)

Trial-by-trial estimates of the individual parameters (*V*_*t*+1_, *SV*_*t*+1_, *k*) are obtained by the marginalization of the joint probability function. For more details, see Behrens and colleagues (2007).

### Image acquisition and preprocessing

MRI data were acquired with a Siemens Trio 3T scanner. Both the fMRI and high-resolution 3D structural brain data were obtained using a 64-channel phased-array head. The fMRI data were acquired by means of a gradient-echo echo-planar imaging sequence containing the following parameters: repetition time (TR) = 1,000 ms, echo time (TE) = 30 ms, 78 multiband slices, voxel size = 2 × 2 × 2 mm [3], flip angle = 35°, field of view (FOV) = 192 mm × 192 mm, data matrix = 96 × 96, and 240 volumes scanned in 2,640 seconds. Additionally, the 3D structural brain images (1 mm [3] isotropic) were acquired for each participant using a T1-weighted 3D magnetization-prepared rapid gradient echo sequence with the following parameters: TR/TE = 2,300 ms/2.26 ms, flip angle = 8°, data matrix = 232 × 256, FOV = 232 mm × 256 mm, BandWidth = 200 Hz/pixel, 192 image slices along the sagittal orientation, obtained in about 9 min.

Functional MRI data were preprocessed with DPABI [39] (http://rfmri.org/dpabi), a software package based on SPM12 (version no.7219; https://www.fil.ion.ucl.ac.uk/spm/software/spm12/). It comprised the following steps: (i) realignment; (ii) co-registering the T1-weighted image to the corresponding mean functional image; (iii) segmenting into gray matter, white matter, and cerebrospinal fluid by DARTEL; (iv) normalizing to the standard Montreal Neurological Institute space (MNI template, resampling voxel size 2 × 2 × 2 mm [3]); (v) smoothing with a Gaussian kernel of 6 mm full width at half maximum (FWHM).

### Generalized linear models (GLM)

SPM12 (version no.7219; https://www.fil.ion.ucl.ac.uk/spm/software/spm12/) was used for general linear model (GLM) analysis. The current study focused on the interaction effect between cue (fear/neut) and volatility (infreq/freq), or fear-biased adaptation to volatility learning, which was defined as [(fear & freq–fear & infreq)–(neut & freq–neut & infreq)].

### Neural responses to learning rates

Based on the winning model and its parameter estimation, the learning rate for each condition (mean value across 4,000 iterations) was obtained. The first model (GLM1) aimed to examine fear-biased adaptation to volatility learning at the neural level in terms of learning rates. The first-level design matrix in GLM1 included the cue for fearful and neutral expressions, option presentation, highlight of the selected options, jitter, and outcome for each condition. If any, we modeled missing trials with option presentation, highlight of the selected options, jitter, and outcome. In addition, the 6 head motion parameters and an FD regressor were included as covariates of no interest. The regressors were then convolved with the canonical hemodynamic response function (HRF). To obtain the fear-biased adaptation to volatility learning effect, we calculated contrasted images [(fear & freq–fear & infreq)–(neut & freq–neut & infreq)] at outcome stage. These contrasted betas further regressed against fear-biased adaptation to volatility learning in terms of learning rate [(fear & freq–fear & infreq)–(neut & freq–neut & infreq)] at the second level.

### Neural responses to subjective volatility

Trial-by-trial subjective volatility from Bayesian Learner model was used to examine neural correlates of fear-biased adaptation to volatility learning in terms of subjective volatility (GLM2). The first-level design matrix in GLM2 included the cue for fearful and neutral expressions, option presentation with the parametric modulation by subjective volatility, highlight of the selected options, jitter with the parametric modulation by subjective volatility, and outcome with the parametric modulation of subjective volatility for each condition. If any, we modeled missing trials with option presentation, highlight, jitter, and outcome. In addition, the 6 head motion parameters and an FD regressor were included as covariates of no interest. The regressors were then convolved with the canonical HRF. Finally, contrasted images [(fear & freq–fear & infreq)–(neut & freq–neut & infreq)] at outcome stage for the modulator effect of subjective volatility from the first-level analysis were entered into one-sample *t*-tests for the second-level analysis.

### Generalized psychophysiological interaction (gPPI)

To assess how the functional connectivity between BOLD signals in the seed region and BOLD signals in the target region was modulated by fear-biased adaptation to volatility learning, we performed gPPI using the gPPI toolbox [40]. The seed region(s) were determined by activation results from GLM2. Specifically, we extracted the deconvolved (with HRF) time course from the first eigenvariate of the seed region (the physiological term) for each participant. The subjective volatility at outcome onset for each condition was used as the psychological term. The interaction term was then generated by multiplying the physiological term with the psychological term. The convolved interaction term was entered into the GLM analysis.

### Dynamic causal modeling (DCM)

To assess effective connectivity between the brain regions underlying the modulation of fear-biased adaptation, we performed DCM using the DCM12 toolbox [41]. We used the same parametric signals (subjective volatility) from each condition to test fear-biased adaptation effects. Six models were constructed with different assumptions of modulatory effects (B matrix) and driving effects (C matrix) while we fixed the full intrinsic connectivity (A matrix; Fig 4A). For the winning model, ANOVAs were performed with cue (fear/neut) and volatility (freq/infreq) as within-subject factors in A, B, and C matrices, respectively. Bonferroni correction was used to correct for multiple comparisons.

### Supplementary experiments

To test the influences of fear on punishment learning, we conducted an additional experiment with the design of 2 (fear/neut) by 2 (freq/infreq) in the punishment context (expS1; *n* = 27). To control for potential impact of attention-grabbing (rather than experiencing fear) on adaptation to volatility, we conducted a control experiment (expS2; *n* = 39). The design was 2 (happy versus neutral facial expressions) by 2 (freq/infreq). Happy expressions were used here because fearful and happy expressions have similar properties of attention-grabbing but distinct affective experience [38]. To test the validity of fear induction (i.e., presentation of fearful/neutral facial expressions before each trial), we collected post-rating data for the presented facial expressions of “how fearful do you feel when seeing this face” using a Likert scale from 0 (no experience of fear at all) to 8 (strong experience of fear) following expS1 and expS2 in S4 Text.

## Supporting information

S1 Text Statistic values for main effects and interaction effects. (DOCX)

S2 Text Supplementary behavioral analysis. (DOCX)

S3 Text Supplementary neuroimaging analysis. (DOCX)

S4 Text Supplementary experiments. (DOCX)

S5 Text Validation of fear induction. (DOCX)

S1 Fig Correlations of learning rates among each condition in exp1 and exp2. The source data can be found at https://osf.io/avhne/.
(TIF)

S2 Fig Identification ratings for fearful and neutral expressions. Data are represented as mean (SE). Note: SE, standard error; *p < 0.05. The source data can be found at https://osf.io/avhne/.
(TIF)

S3 Fig Fear experience ratings for fearful-neutral and neutral-happy expressions. Data are represented as mean (SE). Note: SE, standard error; *p < 0.05. The source data can be found at https://osf.io/avhne/.
(TIF)

S4 Fig Model validation using the correlation between real accuracy and simulated accuracy. The source data can be found at https://osf.io/avhne/.
(TIF)

S5 Fig Model validation using the correlation between real and simulated win-stay loss-switch behavior (WSLS). Note that WSLS sums win-stay rate and loss-switch rate. The source data can be found at https://osf.io/avhne/.
(TIF)

S6 Fig Parameter recovery. The source data can be found at https://osf.io/avhne/.
(TIF)

S7 Fig Win-stay loss-switch behavior. Data are represented as mean (SD). Simulated data (hollow circles) from the winning model (M1) showed a similar patter with real data. The source data can be found at https://osf.io/avhne/.
(TIF)

S8 Fig Accuracy in the third trial after reversal. Data are represented as mean (SD). Simulated data (hollow circles) from the winning model (M1) showed a similar patter with real data. Note: *., *p* < 0.05. The source data can be found at https://osf.io/avhne/.
(TIF)

S9 Fig Learning rates from M5 in exp2. The line within the violin plot represents the median value. Note: n.s., not significant; *p < 0.05. The source data can be found at https://osf.io/avhne/.
(TIF)

S10 Fig Learning rate results from the winning model in expS1. The line within the violin plot represents the median value. Note: *p < 0.05. The source data can be found at https://osf.io/avhne/.
(TIF)

S11 Fig Learning rate results from the winning model in expS2. The line within the violin plot represents the median value. Note: n.s., *p* > 0.05. The source data can be found at https://osf.io/avhne/.
(TIF)

S12 Fig Subjective volatility estimated from Bayesian Learner model in an example participant. Solid lines in blue represent trial-by-trial estimated volatility. Dash lines represent reward schedules. The source data can be found at https://osf.io/avhne/.
(TIF)

S13 Fig BOLD regression differences for volatility at the time of the outcome between infrequent and frequent reversals in neutral (A) and fearful (B) conditions. All activations were whole-brain corrected with the threshold of *p* < 0.001 at the voxel level and with the threshold of *p* < 0.05 at the cluster level using family-wise error (FWE) procedure. The source data can be found at https://osf.io/avhne/.
(TIF)

S14 Fig BOLD regression for outcome (win/no win) at the time of the outcome in learning rates-related analysis. All activations were whole-brain corrected with the threshold of *p* < 0.001 at the voxel level and with the threshold of *p* < 0.05 at the cluster level using family-wise error (FWE) procedure. The source data can be found at https://osf.io/avhne/.
(TIF)

S15 Fig BOLD regression for outcome (win/no win) at the time of the outcome in volatility-related analysis. All activations were whole-brain corrected with the threshold of *p* < 0.001 at the voxel level and with the threshold of *p* < 0.05 at the cluster level using family-wise error (FWE) procedure. The source data can be found at https://osf.io/avhne/.
(TIF)

S16 Fig BOLD regression for expected value (Q-value) at the time of the cue using finite impulse filter (FIR) with ROI-based analysis. Data are represented as mean (SE). Note: ***p < 0.001; *p < 0.05. The source data can be found at https://osf.io/avhne/.
(TIF)

S17 Fig Other correlations. The source data can be found at https://osf.io/avhne/.
(TIF)

S1 Table Normative rating of fearful and neutral facial expressions from the Taiwanese Facial Expression Image Database (TFEID). (DOCX)

S2 Table Descriptive data of post-ratings and task performance. (DOCX)

S3 Table Model recovery: model comparison for simulated data form M1. (DOCX)

S4 Table Model comparison for expS1. (DOCX)

S5 Table Model comparison for expS2. (DOCX)

S6 Table Model comparison between the winning model (M1) and M13 assuming no effect of volatility (a learning rate and a decision parameter for each type of cue). (DOCX)

S7 Table Behavioral and BOLD responses in each experimental condition. (DOCX)

## Abbreviations

AA: anxious arousal
AD: anhedonia depression
BMS: Bayesian model selection
BVAQ: Bermond–Vorst Alexithymia Questionnaire
dACC: dorsal anterior cingulate cortex
DCM: dynamic causal modeling
FD: frame-wise displacement
fMRI: functional magnetic resonance imaging
FOV: field of view
FWHM: full width at half maximum
GDA: general distress: anxiety
GDD: general distress: depression
GLM: general linear model
gPPI: generalized psychophysiological interaction
HDI: high density interval
HI: hippocampus
HRF: hemodynamic response function
LMM: linear mixed-effect model
LOOIC: leave-one-out information criterion
MASQ: Mood and Anxiety Symptoms Questionnaire
MCMC: Markov chain Monte Carlo
OFC: orbitofrontal cortex
PPC: posterior parietal cortex
ROI: region of interest
RW: Rescorla–Wagner
SVC: small volume correction
TFEID: Taiwanese Facial Expression Image Database
TPJ: temporal-parietal junction
VS: ventral striatum
WAIC: widely applicable information criterion
