# Supplementary material for: Neurocomputational mechanisms underlying fear-biased adaptation learning in changing environments
Source: PLoS Biol. 2023 May 1;21(5):e3001724. doi: 10.1371/journal.pbio.3001724 (PMC10174591; doi:10.1371/journal.pbio.3001724)
Supplement: S1 Text — (DOCX) [file pbio.3001724.s001.docx]

Regarding learning rates in exp1, we observed a significant interaction effect between cue and environmental volatility (F = 19.095, *p* < 0.001, partial $\eta^{2}$ = 0.24). No significant main effects were found (main effect of cue: F = 0.349, partial $\eta^{2}$ < 0.001; main effect of environmental volatility: F = 0.569, partial $\eta^{2}$ < 0.001). Simple effect analysis showed a higher learning rate for environments with frequent vs. infrequent reversals (F = 13.126, *p* = 0.004, partial $\eta^{2}$ = 0.18), while a reversal pattern was observed when cued by fearful facial expressions (F = 6.538, *p* = 0.079, partial $\eta^{2}$ = 0.10). Another direction for this interaction effect between cue and volatility showed a lower learning rate for fearful vs. neutral cues in the frequent reversal environments (F = 12.306, *p* = 0.005, partial $\eta^{2}$ = 0.17), while a higher learning rate for fearful vs. neutral cues in the infrequent reversal environments (F = 7.140, *p* = 0.058, partial $\eta^{2}$ = 0.11).

With regard to learning rates in exp2, we observed a significant main effect of volatility (F = 3.954, *p* = 0.049, partial $\eta^{2}$ = 0.03; freq > infreq). We also found a significant interaction effect between cue and environmental volatility (F = 22.509, *p* < 0.001, partial $\eta^{2}$ = 0.16). Simple effect analysis showed a higher learning rate for environments with frequent vs. infrequent reversals (F = 22.667, *p* < 0.001, partial $\eta^{2}$ = 0.16), while a comparable pattern was observed when cued by fearful facial expressions (F = 3.799, *p* = 0.322, partial $\eta^{2}$ = 0.03). Another direction for this interaction effect between cue and volatility showed a lower learning rate for fearful vs. neutral cues in the frequent reversal environments (F = 16.859, *p* < 0.001, partial $\eta^{2}$ = 0.13), while a higher learning rate for fearful vs. neutral cues in the infrequent reversal environments (F = 6.776, *p* = 0.063, partial $\eta^{2}$ = 0.06). No significant main effect of cue was found (F = 1.130, *p* = 0.290, partial $\eta^{2}$ < 0.001).

To identify whether the manipulated variables (volatility and cue) influence learning rates in control of inverse temperature, we also added inverse temperature as the covariate in the regression model (LMM; learning rates as the dependent variables). We still found a significant interaction effect between cue and environmental volatility in each experiment (exp1: F = 18.570, *p* < 0.001, partial $\eta^{2}$ = 0.27; exp2: F = 20.632, *p* < 0.001, partial $\eta^{2}$ = 0.15). Simple effect analysis showed a higher learning rate for environments with frequent vs. infrequent reversals (exp1: F = 12.838, *p* = 0.004, partial $\eta^{2}$ = 0.18; exp2: F = 18.714, *p* < 0.001, partial $\eta^{2}$ = 0.13), while this pattern was disappeared or reversed when cued by fearful facial expressions (exp1: F = 6.477, *p* = 0.081, partial $\eta^{2}$ = 0.10; exp2: F = 4.046, *p* = 0.278, partial $\eta^{2}$ = 0.03). No significant main effects was found (exp1: *ps* >0.470; exp2: *ps* > 0.090). We also checked whether the manipulated variables (volatility and cue) influence inverse temperature in control of learning rates. LMM was conducted with cue and volatility as within-subject factors, with subject as random factor, and with learning rates as a regressor on inverse temperature. No significant interaction effect was found in neither exp1 nor exp2 (*ps* >0.125). These results suggest that the manipulated variables specifically impacted learning rates.

In terms of parametric effects of subjective volatility in the dACC, we observed a significant interaction effect between cue and environmental volatility (F = 14.609, *p* < 0.001, partial $\eta^{2}$ = 0.29). Simple effect analysis showed significant increases in infreq versus freq in neutral cues (F = 13.272, *p* = 0.001, partial *η^2^* = 0.27). However, this pattern disappeared for fearful cues (F = 1.833, *p* = 0.184, partial *η^2^* = 0.05). No significant main effects was found (main effect of cue: F = 1.763, *p* = 0.193, partial $\eta^{2}$ = 0.05; main effect of environmental volatility: F_1,36_ = 0.933, partial $\eta^{2}$ = 0.025). As for parametric effects of subjective volatility in the VS, we observed a significant interaction effect between cue and environmental volatility (F = 15.274, *p* < 0.001, partial $\eta^{2}$ = 0.30). Simple effect analysis showed significant increases in infreq versus freq in neutral cues (F = 8.191, *p* = 0.007, partial *η^2^* = 0.19). However, this pattern disappeared for fearful cues (F = 3.966, *p* = 0.054, partial *η^2^* = 0.10). No significant main effects was found (main effect of cue: F = 0.021, partial $\eta^{2}$ = 0.00; main effect of environmental volatility: F = 0.090, partial $\eta^{2}$ = 0.00).

For functional connectivity between the dACC and TPJ, we observed a significant interaction effect between cue and environmental volatility (F = 26.551, *p* < 0.001, partial $\eta^{2}$ = 0.42). Simple effect analysis showed that functional connectivity between dACC and TPJ increased in infreq as compared to freq following neutral cues (F = 11.306, *p* = 0.002, partial *η^2^* = 0.24), but decreased following fearful cues (F = 4.748, *p* = 0.036, partial *η^2^* = 0.12). No significant main effects were found (main effect of cue: F = 0.172, partial $\eta^{2}$ = 0.01; main effect of environmental volatility: F= 0.455, partial $\eta^{2}$ = 0.01).

Regarding the driving effect on the TPJ, we found a significant main effect of cue (F = 8.321, *p* = 0.007, partial $\eta^{2}$ = 0.19; fear > neut) and a significant interaction effect between cue and environmental volatility (F = 8.321, *p* = 0.007, partial $\eta^{2}$ = 0.19). Simple effect analysis showed a significant increase in infreq than freq in neutral cues (F= 9.827, *p* = 0.003, partial *η^2^* = 0.21), but not fearful cues (F = 0.064, partial *η^2^* = 0.00). No significant main effect of environmental volatility was observed (F = 2.476, *p* = 0.124, partial $\eta^{2}$ = 0.06). Given that the winning model (DCM model 3) was close to model 2, as shown by exceedance probability for model 3 (about 0.6) and model 2 (about 0.3; Figure 4C), we also checked differences of the driving effect on the TPJ. An ANOVA of 2 (fear/neut) by 2 (freq/infreq) showed a significant main effect of cue (F = 8.321, *p* = 0.007, partial $\eta^{2}$ = 0.19; fear > neut) and a significant interaction effect between cue and environmental volatility (F = 8.321, *p* = 0.007, partial $\eta^{2}$ = 0.19). Simple effect analysis showed a significant increase in infreq than freq in neutral cues (F= 9.827, *p* = 0.003, partial *η^2^* = 0.21), but not fearful cues (F = 0.064, partial *η^2^* = 0.00). No significant main effect of environmental volatility was observed (F = 2.476, *p* = 0.124, partial $\eta^{2}$ = 0.06).
