# Supplementary material for: Neurocomputational mechanisms underlying fear-biased adaptation learning in changing environments
Source: PLoS Biol. 2023 May 1;21(5):e3001724. doi: 10.1371/journal.pbio.3001724 (PMC10174591; doi:10.1371/journal.pbio.3001724)
Supplement: S2 Text — (DOCX) [file pbio.3001724.s002.docx]

**Reaction time (RT)**

We checked RT in each condition. A 2 (fear/neutral) by 2(freq/infreq) ANOVA showed no significant effect in exp1 (*ps* >0.238) and exp2 (*ps* > 0.147). Consistent with previous studies using reversal learning tasks(Crawley et al., 2020), these results suggest that choice data are more sensitive to capture the psychological characteristics of participants during this learning processing.

**Accuracy**

Regarding accuracy, we conducted a 2 (fear/neut) by 2(freq/infreq) ANOVA in exp1 and exp2. Results showed a significant main effect of volatility (exp1: F = 6.117, *p* = 0.022, partial $\eta^{2}$ = 0.23, freq<infreq; exp2: F = 18.403, *p* < 0.001, partial $\eta^{2}$ = 0.32, freq<infreq). No other significant effect was found (exp1: *ps* >0.228; exp2: *ps* > 0.059). These results were consistent with previous volatility-related literature(Liu et al., 2022), suggesting that the frequently changed environments are more difficult to learn as compared to the infrequent counterpart.

**Performance after reversal**

It has been shown that performance after reversal reflects changes in learning rates(Piray et al., 2019). Therefore, we checked how conditional differences in learning rates reflect participants’ performance after reversal. To avoid insufficient power, we merged data from exp1 and exp2 (n=61). Firstly, one-sample *t*-test (as compared to 0.5) showed that accuracy (i.e., the proportion to select the optimal option) in the 1^st^ trial after reversal for all conditions were significantly lower than the random level (i.e., 0.5; *ps* < 0.008). This suggests that participants were able to learn the reward structure of this task. More importantly, an ANOVA of 2(fear/neut) by 2(freq/infreq) in accuracy for each trial after reversal showed a significant interaction effect between cue and volatility for the 3^rd^ trial after reversal (F = 13.566, *p* < 0.001, partial $\eta^{2}$ = 0.18; FDR corrected; S8_Fig). Simple effect analysis showed higher accuracy for freq vs. infreq in face of neutral cues (F = 9.622, *p* = 0.003, partial $\eta^{2}$ = 0.14). Given that a higher learning rate generally makes people adapt to reversal fast, the finding of better performance after reversal for environments with frequent vs. infrequent reversals may result from the observed higher learning rate for freq than infreq condition. However, a reversal pattern was observed when cued by fearful facial expressions (F = 4.291, *p* = 0.043, partial $\eta^{2}$ = 0.07), suggesting that fear disrupts reversal adaptation. This is consistent with learning rates pattern under fear, indicating that abnormal learning speed in face of fear interferes reversal adaptation. Data recovery also confirmed this pattern: the significant interaction effect between cue and volatility (F = 13.346, *p* = 0.001, partial $\eta^{2}$ = 0.18; S8_Fig), with higher accuracy for environments with frequent vs. infrequent reversals in face of neutral cues (F = 10.357, *p* = 0.002, partial $\eta^{2}$ = 0.15), but not in the fearful condition (F = 2.101, *p* = 0.152, partial $\eta^{2}$ = 0.03). In sum, these results showed the same pattern with conditional differences in learning rates, suggesting that atypical mental computations for adaptation learning under fear result in aberrant learning performance, especially after reversal.

**Win-stay loss-switch behavior**

Concerning win-stay and loss-stay behavior, we performed an ANOVA of 2(win/loss) by 2(fear/neut) by 2(freq/infreq) in choice proportion in exp1and exp2. Results showed a significant main effect of win/loss (F = 79.213, *p* < 0.001, partial $\eta^{2}$ = 0.80, win-stay>loss-stay, S7A_Fig; exp2: F = 115.751, *p* < 0.001, partial $\eta^{2}$ = 0.75, win-stay>loss-stay, S7C_Fig). No other significant effect was found (exp1: *ps* > 0.254; exp2: *ps* > 0.097). We also used win-stay loss-switch behavior (WSLS; sum win-stay ratio and loss-switch ratio up) to represent learning behavior. An ANOVA of 2(fear/neut) by 2(freq/infreq) in WSLS showed no significant effect (exp1: *ps* > 0.553; exp2: *ps* > 0.570). Combining with our finding of fear-biased adaptation to volatility in terms of learning rates and of performance after reversal, these results together suggest that the psychological characteristics of participants during this learning processing, as captured by mental parameters, are more sensitive to performance after reversal.

**Parameter and model recovery**

Due to huge computational costs for the Markov chain Monte Carlo (MCMC) fitting method and for simplicity, we performed parameter and model recovery analyses only for the winning model (M1). We simulated data from each participant in both exp1 and exp2. After fitting these simulated data using hierarchical Bayesian estimation with MCMC, M1 was the winning model, in addition to M3 in exp1 (slight difference between M1 and M3: ΔLOOIC=-20.1, ΔWAIC=-14.1; S3_ Table). Overall, M1 was identifiable. Parameter recovery analysis showed high correlations between real and simulated parameters (exp1: *rs* > 0.524, *ps* < 0.015, in addition to r = 0.335, *p* = 0.137 in learning rate parameter in the fear & infreq condition; exp2: *rs* > 0.703, *ps* < 0.001; S6_Fig). Given one insignificant value (*p* = 0.137) above-mentioned, we further performed correlation analysis between recovered learning rates-bias with alexithymia. We observed same pattern with the real parameter (BVAQ: r = 0.283, *p* = 0.027; BVAQ-C: r = 0.268, *p* = 0.037; BVAQ-A: r = 0.142, *p* = 0.275). We also ran GLM1 of brain activation with recovered learning rates-bias as a regressor to check the robustness of parameter recovery. We found significant activations in the VS (peak at [4 8 -6], r = 0.512, *p* = 0.001, k = 3) and HI (peak at [-30 -18 -22], r = 0.575, *p* < 0.001, k = 14). These results showed that parameters were recovered well from the winning model. Please note that the current model-related step-by-step strategy should not bias toward M1. Specifically, we first performed model comparison to select the winning model. This selection procedure ensured that the winning model fit our data best among our model space. Then we perform parameter and model recovery to ensure the validity of the winning model. We also conducted posterior predictive check (PPC) to check how well the winning model can capture the real data.

**Fix slope at 1 based on M12**

We examined linear representation model with the slope fixing at 1 (M13) based on M12. As compared to the winning model (M1), M13 performed slight better than M1 in exp1 (ΔLOOIC=+2.8, ΔWAIC=+5.8), whereas M13 performed poorly in exp2 (ΔLOOIC=-4634.1, ΔWAIC=-115839.6). Overall, M1 fit data better. In addition, we used LOOIC and WAIC to perform model comparison, which penalized model complexity. Therefore, the approach allowing the slope to vary can avoid parameter saturation to some extent.

**Control analysis for alexithymia**

Anxiety and depression has been demonstrated to co-occur with alexithymia(Hendryx et al., 1991; Li et al., 2015). We, therefore, performed partial correlations to control for the potential influences of anxiety and depression, as in our previous studies(Wang, Chen, et al., 2021; Wang, Goerlich, et al., 2021). Correlation coefficients remained significant for total BVAQ scores and cognitive dimensional scores (*rs* > 0.319, *ps* < 0.015). For exploratory purposes, we also checked correlations of behavioural bias with anxiety- and depression-related questionnaires. No significant correlation was found (*ps* > 0.487). On the other hand, there are six subscales for the Chinese version of BVAQ(Wang, Wang, et al., 2021). Recently, it has been demonstrated that measuring alexithymia with the sum score for each dimension is more reliable(Carnovale et al., 2021). Therefore, we focused on the high-order cognitive and affective structures in BVAQ measurements, which has been shown good psychometric properties in our previous study(Wang, Wang, et al., 2021). In sum, individuals prone to cognitive alexithymia were more influenced by fear in adaptation to volatility.
