# Supplementary material for: Neurocomputational mechanisms underlying fear-biased adaptation learning in changing environments
Source: PLoS Biol. 2023 May 1;21(5):e3001724. doi: 10.1371/journal.pbio.3001724 (PMC10174591; doi:10.1371/journal.pbio.3001724)
Supplement: S3 Text — (DOCX) [file pbio.3001724.s003.docx]

**Replication of the previous finding in dACC**

It has been shown signals from the dACC tracked trial-by-trial subjective volatility(Behrens et al., 2007, 2008). We first checked whether our data can replicate their finding in our control condition. Within the previously reported location dACC (MNI: x = -6, y = 26, z = 34, a sphere with 10mm radium) from Behrens.et.al., (2007), we found a significant activation for subjective volatility in the dACC (peak at [-2 26 30], k=91; S13_Fig).

**Validation of activation in the dACC with different sphere radiuses.**

In addition to 10mm sphere in the dACC, we validated the dACC’s result with 8mm and 12mm sphere radiuses. Our results were significant (8mm: peak at [-2 32 32], k=2; 12mm: peak at [-2 32 32], k=9), suggesting the robustness of the dACC activation to encode subjective volatility.

**Robust for the PPC result**

We identified an outlier (±3SD) in Figure 3A. After removing the outlier, the correlation in fear-biased adaptation to volatility between learning rates and activation in the PPC remained significant (r = 0.423, *p* = 0.010).

**Voxel-wise and cluster-wise thresholds**

Our neural findings were corrected with the threshold of *p* < 0.001 at the voxel level and with the threshold of *p* < 0.05 at the cluster level using AlphaSim procedure. Using a stricter correction method family-wise error (FWE), all neural results hold in addition to the PPC. Therefore, the interpretation for the PPC finding should be cautious.

**Control for outcome signals**

We validated our neuroimaging results by controlling for potential differences in outcome (i.e., including win/no win as a regressor on outcome onsets). For learning rates-related signals, we first replicated our findings in the PPC (whole-brain analysis, peak at [-50 -60 50], k=108), HI (ROI analysis, peak at [28 -16 -22], k = 10), and VS (ROI analysis, peak at [4 8 -4], k = 6) in response to fear-biased adaptation to volatility. Based on the previous meta-analysis on valuation(Bartra et al., 2013), we selected striatum and medial prefrontal cortex (mPFC; from AAL atlas) for ROI analyses. Regarding outcome signals per se, we confirmed significant activations in the striatum (cluster 1: peak at [10 8 -8], k = 195; cluster 2: peak at [-12 6 -10], k = 250; cluster 3: peak at [-30 -8 0], k = 102; cluster 4: peak at [32 -8 0], k = 86) and mPFC (peak at [4 40 -10], k = 1415; S14_Fig).

For subjective volatility-related signals, we replicated our findings in the dACC (peak at [-2 34 32], k = 8) and VS (peak at [10 16 -8], k = 11). We also confirmed significant activations in the striatum (cluster 1: peak at [-30 -8 2], k = 1162; cluster 2: peak at [20 10 -10], k = 874; cluster 3: peak at [18 6 18], k = 310) and mPFC (peak at [8 44 -2], k = 440; S15_Fig). These results suggest that our neural findings of fear-biased adaptation to volatility is robust.

**Signals of expected value**

Based on the GLM2 (neural signal for volatility), we examined brain activation of expected value (Q-value) using finite Impulse Response (FIR). Specifically, we added expected value as parametric modulator on cue onsets and estimated 10 time-points over a window of 10 seconds (TR=1s). Based on the previous meta-analysis of valuation^12^, we selected the striatum and medial prefrontal cortex (mPFC; from AAL atlas) for ROI analyses. We found that significant activation in the striatum bumped in the 3^rd^ (peak at [-26 -10 4], k = 9, S16A_Fig), 4^th^ (peak at [16 12 10], k =22, S16B_Fig), and 5^th^ (peak at [18 16 6], k = 14, S16C_Fig) points. However, we did not observe the significant activation in the mPFC. One potential explanation is low signal-noise ratio for the mPFC BOLD signals(Deichmann et al., 2003). Overall, we replicated previous findings of Q-value signals.
