# Supplementary material for: Neurocomputational mechanisms underlying fear-biased adaptation learning in changing environments
Source: PLoS Biol. 2023 May 1;21(5):e3001724. doi: 10.1371/journal.pbio.3001724 (PMC10174591; doi:10.1371/journal.pbio.3001724)
Supplement: S4 Text — (DOCX) [file pbio.3001724.s004.docx]

**Fear-biased adaptation to volatility in the punishment context (expS1)**

Another important question is whether our findings of fear-biased adaptation to volatility in the reward context can be generalized to the punishment context. Previous human studies have shown successful adaptation to volatility in both reward and punishment contexts. Specifically, Behrens et al., (2007) found a higher learning rate to seek monetary rewards in the volatile than stable environment. A higher learning rate was also observed to avoid electrical shocks in volatile versus the stable conditions(Browning et al., 2015). The flexible adaptation to volatility can be generalized to other contexts (e.g., social(Behrens et al., 2008)) and other species (e.g., monkeys(Massi et al., 2018)), and other populations (e.g., childhood(Manning et al., 2017)). We also replicated these findings in the condition of neutral facial expression. Therefore, during punishment learning, we hypothesized a successful adaptation to volatility in neutral conditions (e.g., cued by the neutral facial expression). Interestingly, we found that flexible adjustments to volatility in reward contexts were suppressed by fearful facial expressions across two experiments. It has been shown that anxious individuals have difficulties in adaptation to volatility during aversive environments(Browning et al., 2015). Given the similar structure between anxiety and fear(Steimer, 2002), we hypothesized that fear would suppress adaptation to volatility in the punishment context.

We conducted an experiment (exp S1; n = 27, 15 females; age: 22.00±2.08) to test the influences of fear on punishment learning. Specifically, we have designed a 2 (fear/neut) by 2 (freq/infreq) experiment (expS1) in the punishment context. Following the experiment, participants completed fear ratings. Model comparison showed that M12, assuming linear relationship among learning rates, outperformed (mean pseudoR2 = 0.29; mean balanced accuracy = 0.67, which was significantly higher than random level (i.e., 50%, *p*<0.001); S4_Table). Similar to M1, M12 additionally assumed linear relationship among learning rates, except that each condition had a learning rate and a decision parameter. This inconsistency for different winning models in may attribute to the contextual effect (the punishment vs. reward context). We then implemented LMM with subject as a random factor and with cue (fear/neut) and volatility (freq/infreq) as within-subject factors for M12. Results showed a significant interaction effect between cue and volatility (F = 96.043, *p* < 0.001, partial $\eta^{2}$ = 0.55; S10_Fig). Simple effect analysis showed a higher learning rate for environments with freq vs. infreq (F = 8.243, *p* = 0.032, partial $\eta^{2}$ = 0.10, while a reversal pattern was observed when cued by fearful facial expressions (F = 120.758, *p* < 0.001, partial $\eta^{2}$ = 0.61). In addition to the significant main effect of volatility (F = 32.952, *p* < 0.001, partial $\eta^{2}$ = 0.30; freq<infreq), no significant main effect of cue was found (F = 0.046, partial $\eta^{2}$ < 0.001). These findings supported our hypothesis and showed the same pattern with our findings in the reward context, though the punishment context additionally induces linear relationship among learning rates. In sum, these results suggest that fear-biased adaptation to volatility is reward/punishment-independent.

**Control experiment (expS2)**

To control for potential impact of attention-grabbing (rather than experiencing fear) on adaptation to volatility, we conducted a control experiment (expS2; n = 39, 20 females; age: 20.72±2.72). The design was 2 (happy vs. neutral facial expressions) by 2 (freq/infreq). Happy expressions were used here because fearful and happy expressions have similar properties of attention-grabbing but distinct affective experience(Kuppens et al., 2013). We hypothesized that happy cues would not suppress adaptation to volatility. Again, the winning model was M1 (mean pseudoR2 = 0.33; mean balanced accuracy = 0.70, which was significantly higher than random level (i.e., 50%, *p*<0.001); S5_Table). We then implemented linear mixed-effect models (LMM) with subject as a random factor and with cue (happy/neut) and volatility (freq/infreq) as within-subject factors for M1. Results showed no significant interaction effect between cue and volatility (F = 2.623, *p* = 0.108, partial $\eta^{2}$ = 0.02; S11_Fig). No other significant effect was found (*ps* > 0.215). We observed that the learning rate in the frequent condition is slightly lower than that in the infrequent condition in face of neutral expressions (S11_Fig), which did not reach significance (*p* = 0.163). This deviation from our expectation of a higher learning rate for freq vs. infreq in the neutral condition may be attribute to the contextual modulation, which has been shown to influence decision-making and learning(Palminteri, S. et al., 2015). Specifically, when facing happy and neutral expressions, humans perceive the neutral faces as relatively negative, which may further inhibit flexible adjustment, as suggested by our findings that fear disrupted adaptation to volatility. However, interpretation about the insignificant result should be made with cautious. Combining with our findings of fear-biased adaptation to volatility, these results together suggest that fear experience, but not the attentional-distracting property, disrupts flexible adaptation to volatility. It is further worth noting that fixation time on each cue may affect emotional experience (i.e., negative bias), future studies would benefit from physiological measurements (e.g., eye-tracking) to rule out the potential confounding factors on our findings.
