# Supplementary material for: Neurocomputational mechanisms underlying fear-biased adaptation learning in changing environments
Source: PLoS Biol. 2023 May 1;21(5):e3001724. doi: 10.1371/journal.pbio.3001724 (PMC10174591; doi:10.1371/journal.pbio.3001724)
Supplement: S5 Text — (DOCX) [file pbio.3001724.s005.docx]

We collected post-ratings of “To which extent do you think the presented face showed a fearful/neutral expression?” using a Likert scale from 0 (totally not) to 8 (totally agree) following exp1 and exp2. These post-ratings were used to check whether participants correctly recognized stimuli as fearful/neutral expressions. One-sample *t*-tests confirmed that post-ratings were significantly higher than the random level (i.e., 4: midpoint between 0 and 8) both for fearful and neutral expressions (exp1: *ps* < 0.001; exp2: *ps* < 0.001; S2_Fig; S2_ Table), suggesting the participants correctly identified the emotional expressions of the cue.

Although we cannot provide direct information about whether participants feel fearful when seeing fearful facial expressions in exp1 and exp2, the presentation of fearful facial expressions has been thought of as fear induction(Rosenberg & Ekman, 1994; Zhang et al., 2017). The processing of fearful faces has been associated with activation in the amygdala-centric emotional brain systems(Xu et al., 2021). Given the important role of the mirror neuron system (i.e., automatically mimicking the perceived facial expressions) and the empathy networks (e.g., experiencing vicarious feelings) in the perception of emotive facial expression(Bird & Viding, 2014; Wood et al., 2016), the current manipulation of cueing fearful expressions, to some extent, could generate explicit or implicit fearful experience.

To examine whether participants felt fearful when looking at the fearful faces, we collected additional post-rating data for fearful-neutral (expS1) and happy-neutral (expS2) facial expressions of “how fearful do you feel when seeing this face” using a Likert scale from 0 (no experience of fear at all) to 8 (strong experience of fear). Specifically, fearful vs. neutral expressions (expS1) and happy vs. neutral expressions (expS2) were rated, separately. Results showed slight-to-moderate fearful experience when looking at fearful facial expressions (fearful expressions: 3.52±2.32, neutral expressions: 1.85±2.08 in expS1; neutral expressions: 1.96±1.80, happy expressions: 1.12±1.48 in expS2; S3_Fig). We also found stronger fearful experience for fearful than neutral faces, as well as those of neutral compared to happy facial expressions (*ps* < 0.014). Therefore, participants indeed experienced fear during the experiment.

Please note that we presented fearful or neutral faces before each trial rather than each block, because it has been shown that single-trial mood induction (for example, Wheel of Fortune task) only influences the perception of value in initial trials, but not learning speed across the whole experiment(Eldar & Niv, 2015).
