# Supplementary figures and images for: Neurocomputational mechanisms underlying fear-biased adaptation learning in changing environments
Source: PLoS Biol. 2023 May 1;21(5):e3001724. doi: 10.1371/journal.pbio.3001724 (PMC10174591; doi:10.1371/journal.pbio.3001724)

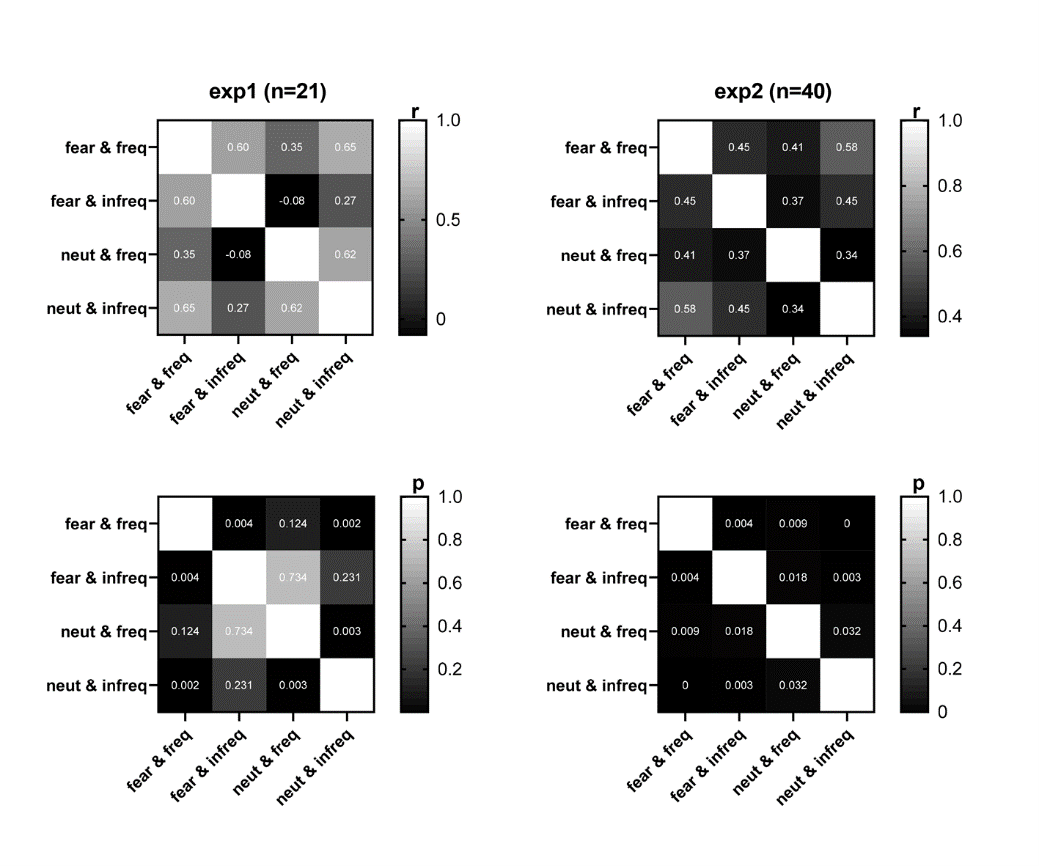

Supplement: S1 Fig — The source data can be found at https://osf.io/avhne/. (TIF) [file pbio.3001724.s006.tif]

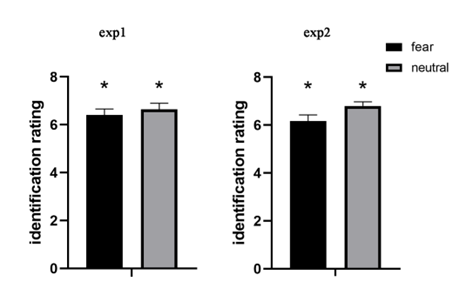

Supplement: S2 Fig — Data are represented as mean (SE). Note: SE, standard error; *p < 0.05. The source data can be found at https://osf.io/avhne/. (TIF) [file pbio.3001724.s007.tif]

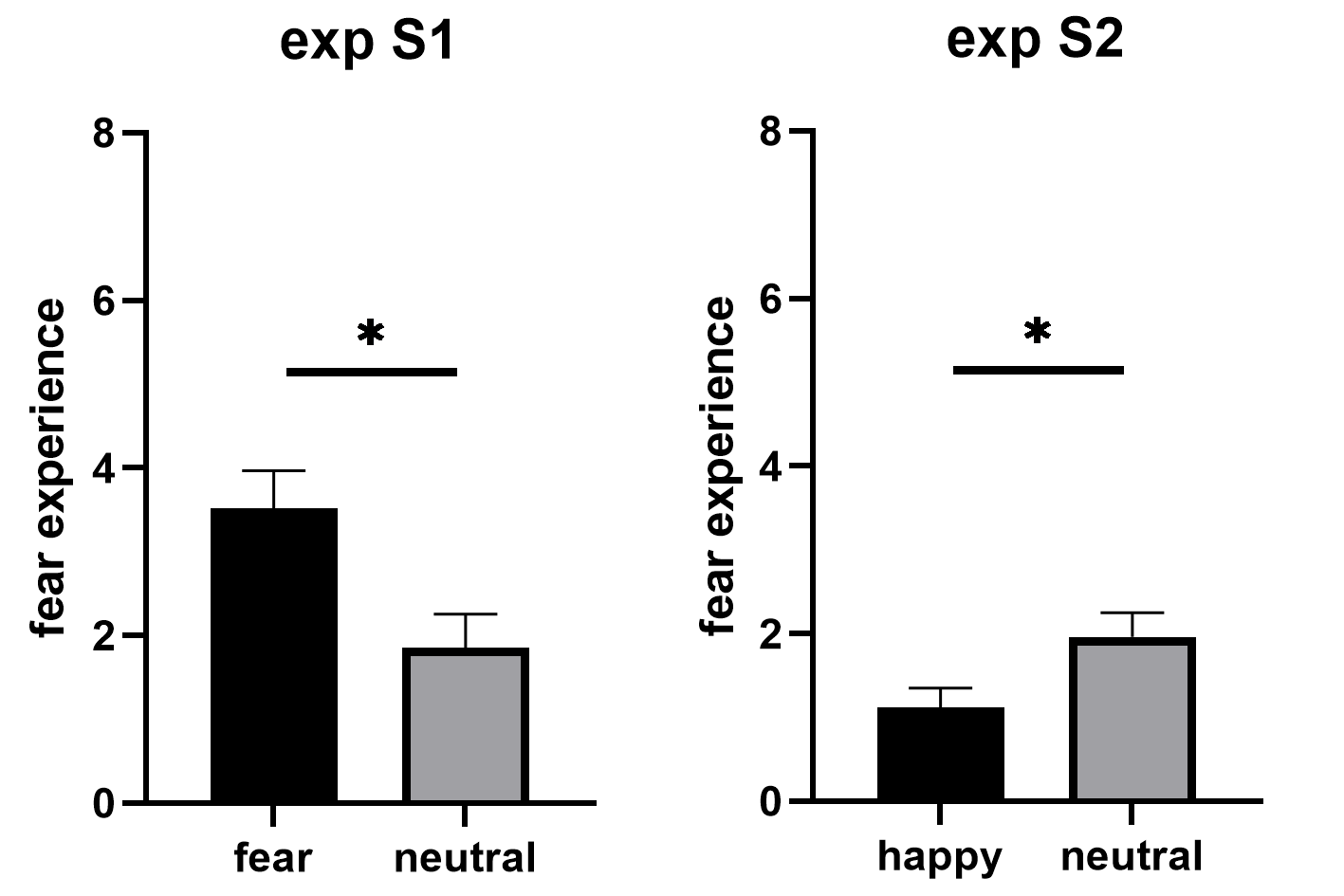

Supplement: S3 Fig — Data are represented as mean (SE). Note: SE, standard error; *p < 0.05. The source data can be found at https://osf.io/avhne/. (TIF) [file pbio.3001724.s008.tif]

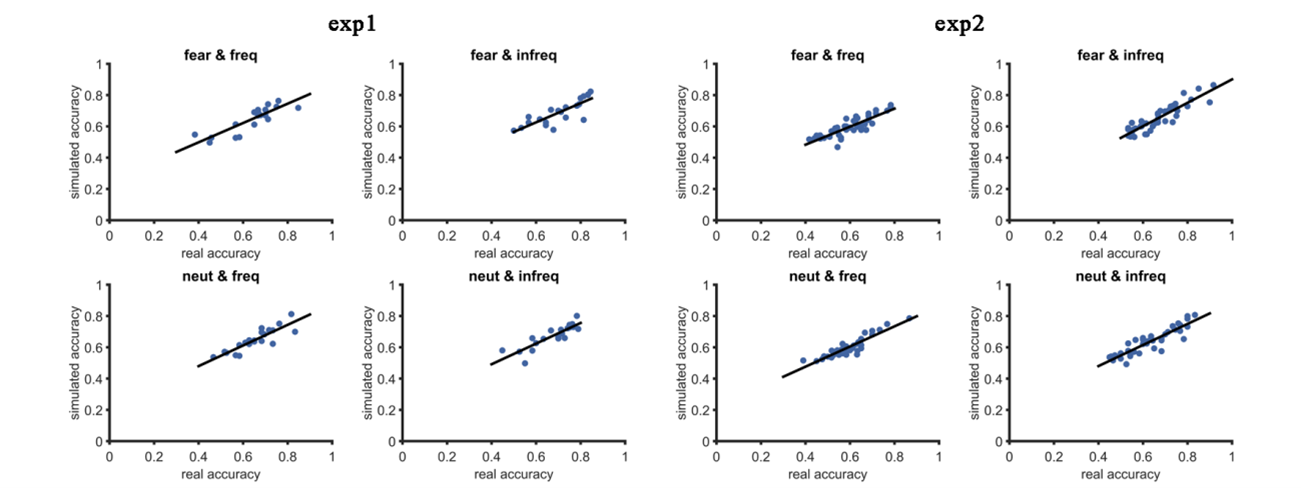

Supplement: S4 Fig — The source data can be found at https://osf.io/avhne/. (TIF) [file pbio.3001724.s009.tif]

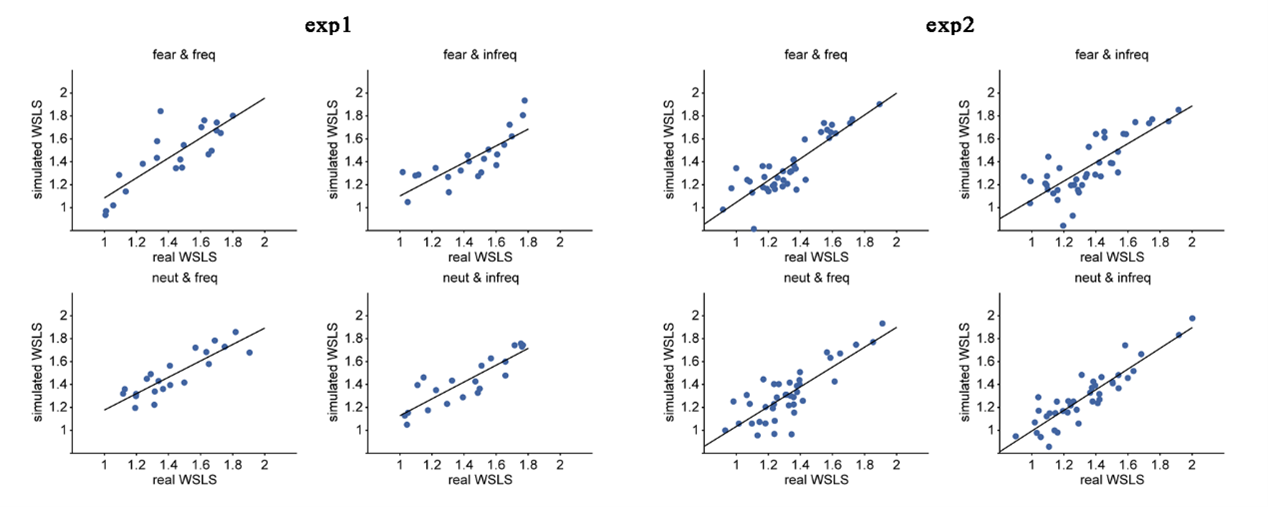

Supplement: S5 Fig — Note that WSLS sums win-stay rate and loss-switch rate. The source data can be found at https://osf.io/avhne/. (TIF) [file pbio.3001724.s010.tif]

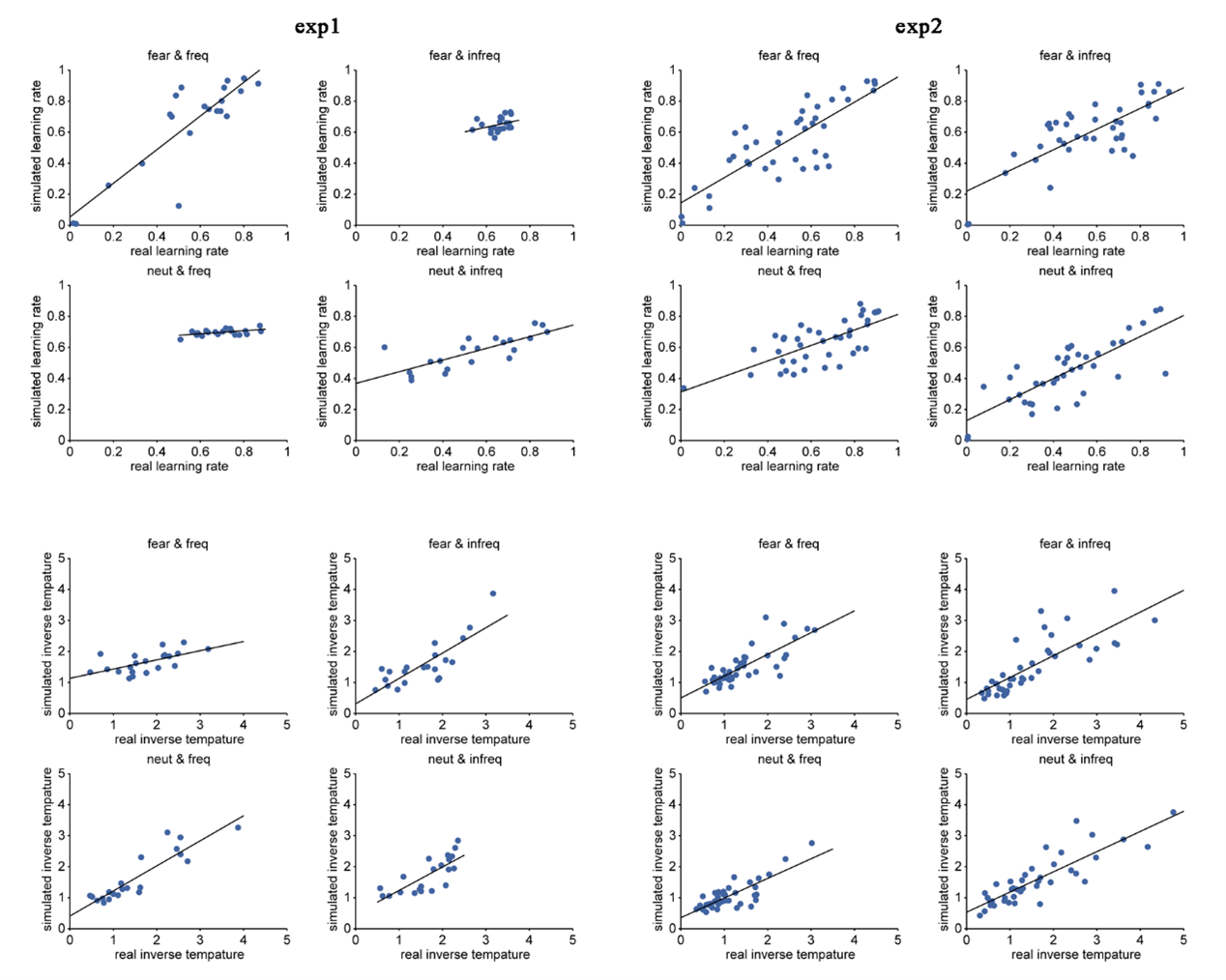

Supplement: S6 Fig — The source data can be found at https://osf.io/avhne/. (TIF) [file pbio.3001724.s011.tif]

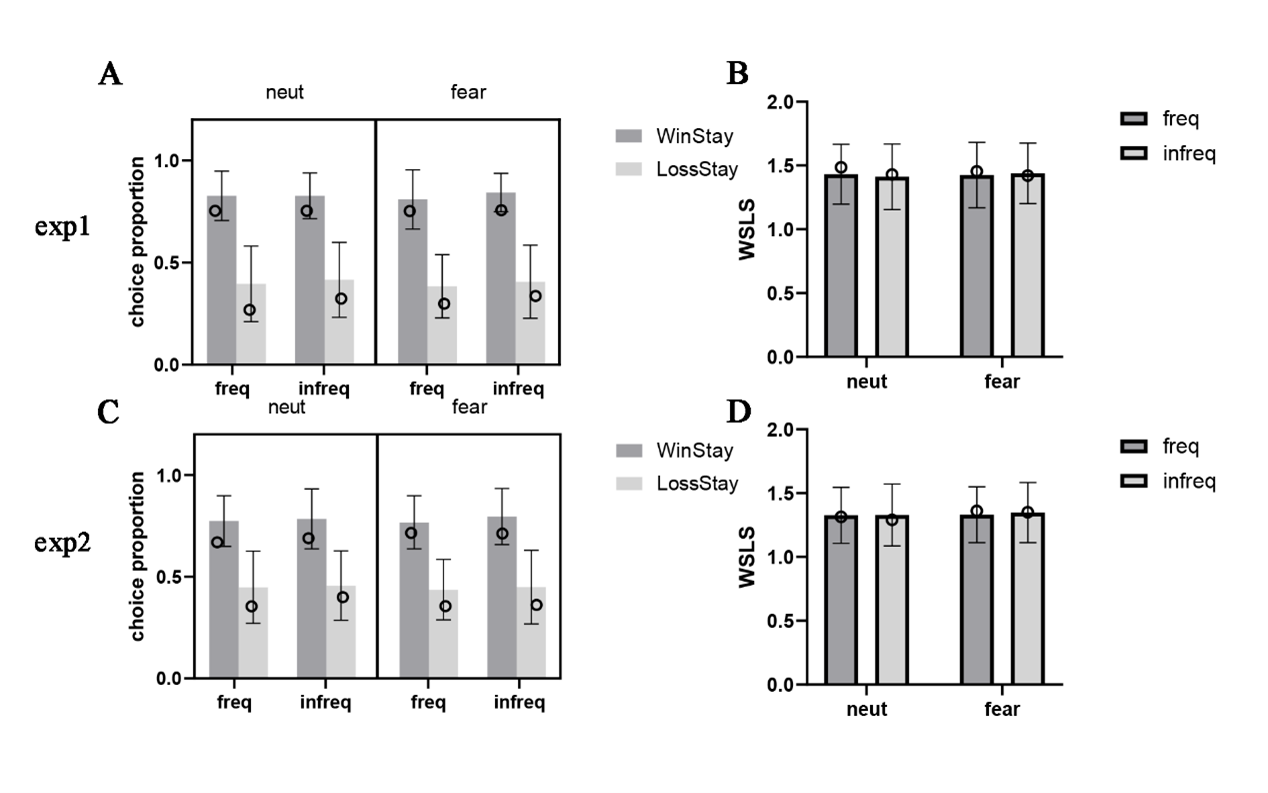

Supplement: S7 Fig — Data are represented as mean (SD). Simulated data (hollow circles) from the winning model (M1) showed a similar patter with real data. The source data can be found at https://osf.io/avhne/. (TIF) [file pbio.3001724.s012.tif]

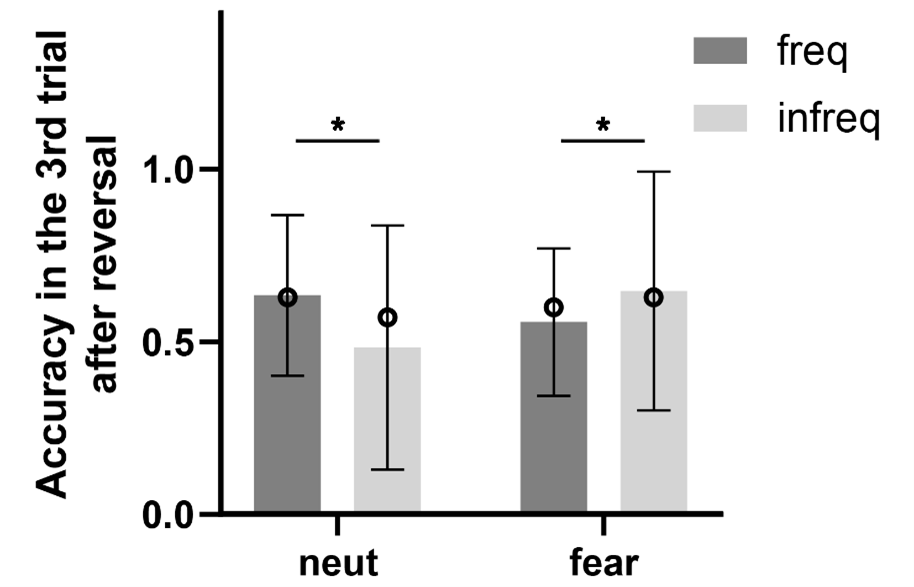

Supplement: S8 Fig — Data are represented as mean (SD). Simulated data (hollow circles) from the winning model (M1) showed a similar patter with real data. Note: *., p < 0.05. The source data can be found at https://osf.io/avhne/. (TIF) [file pbio.3001724.s013.tif]

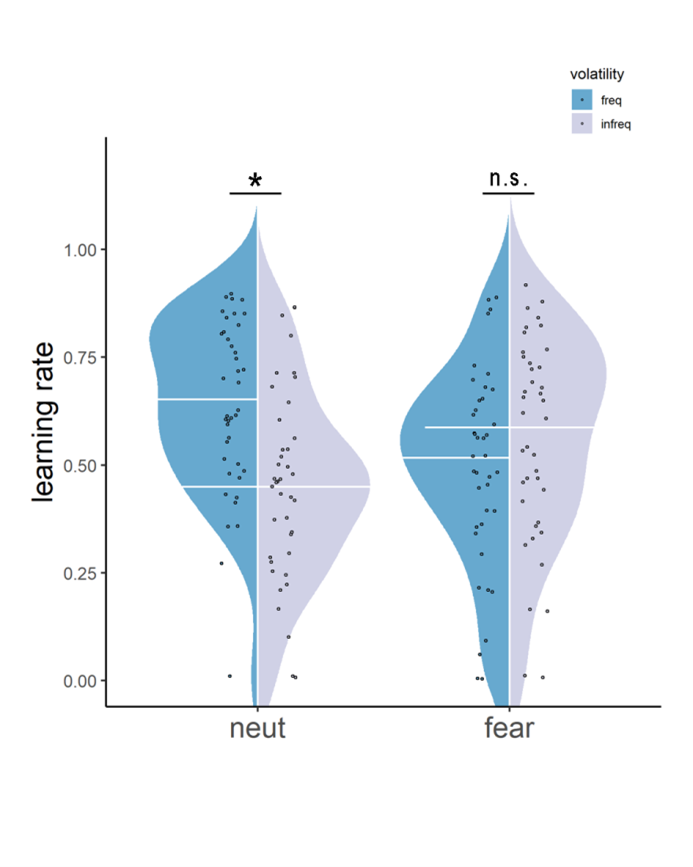

Supplement: S9 Fig — The line within the violin plot represents the median value. Note: n.s., not significant; *p < 0.05. The source data can be found at https://osf.io/avhne/. (TIF) [file pbio.3001724.s014.tif]

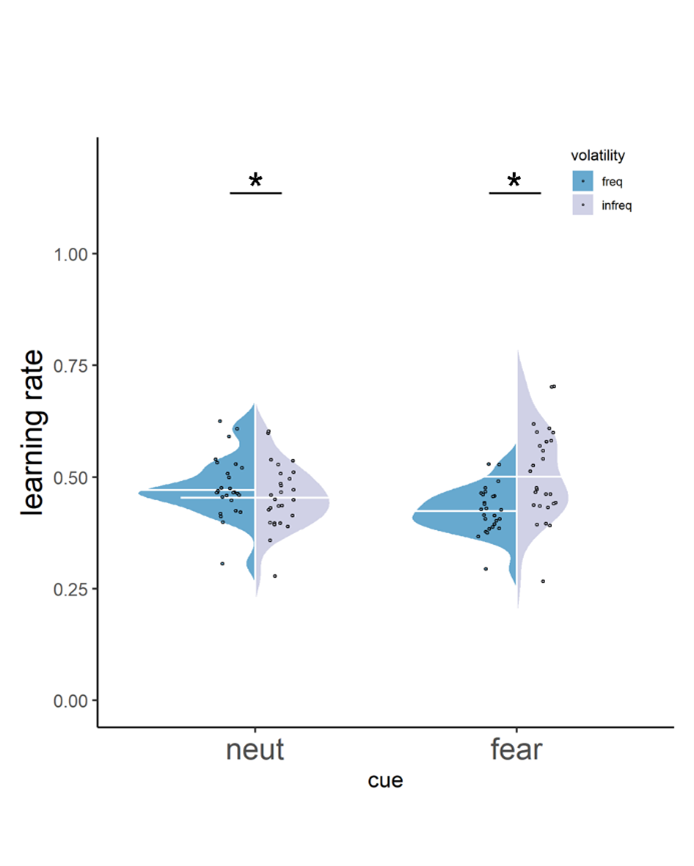

Supplement: S10 Fig — The line within the violin plot represents the median value. Note: *p < 0.05. The source data can be found at https://osf.io/avhne/. (TIF) [file pbio.3001724.s015.tif]

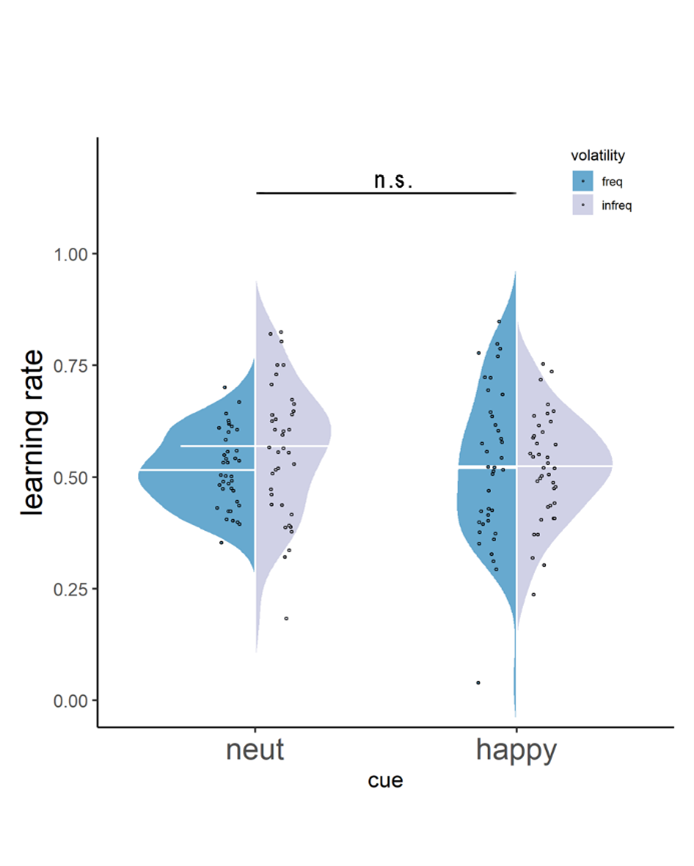

Supplement: S11 Fig — The line within the violin plot represents the median value. Note: n.s., p > 0.05. The source data can be found at https://osf.io/avhne/. (TIF) [file pbio.3001724.s016.tif]

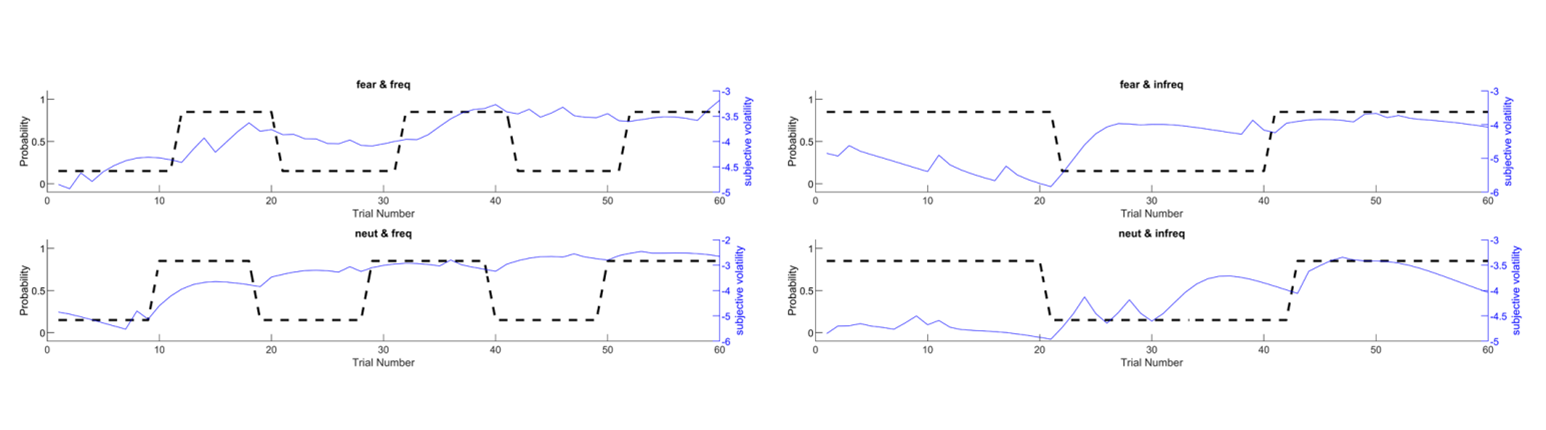

Supplement: S12 Fig — Solid lines in blue represent trial-by-trial estimated volatility. Dash lines represent reward schedules. The source data can be found at https://osf.io/avhne/. (TIF) [file pbio.3001724.s017.tif]

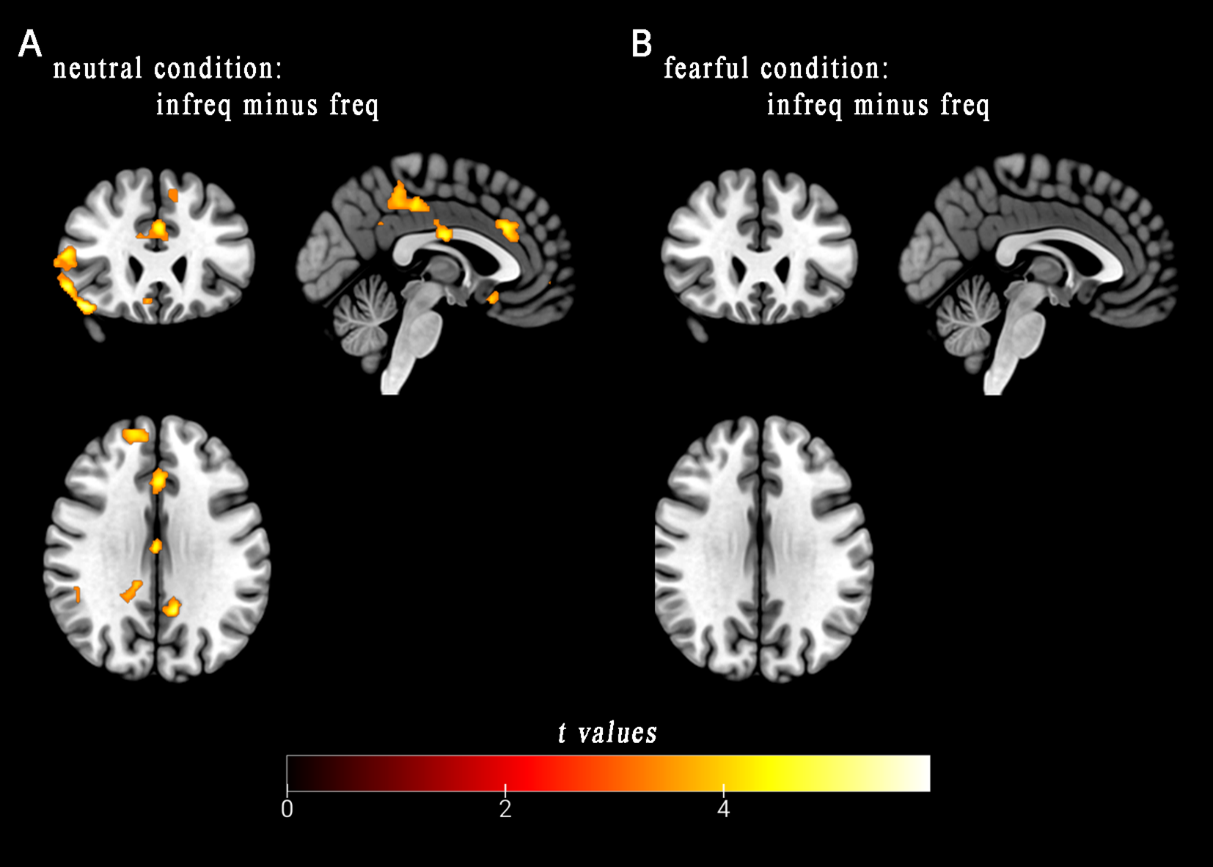

Supplement: S13 Fig — BOLD regression differences for volatility at the time of the outcome between infrequent and frequent reversals in neutral (A) and fearful (B) conditions. All activations were whole-brain corrected with the threshold of p < 0.001 at the voxel level and with the threshold of p < 0.05 at the cluster level using family-wise error (FWE) procedure. The source data can be found at https://osf.io/avhne/. (TIF) [file pbio.3001724.s018.tif]

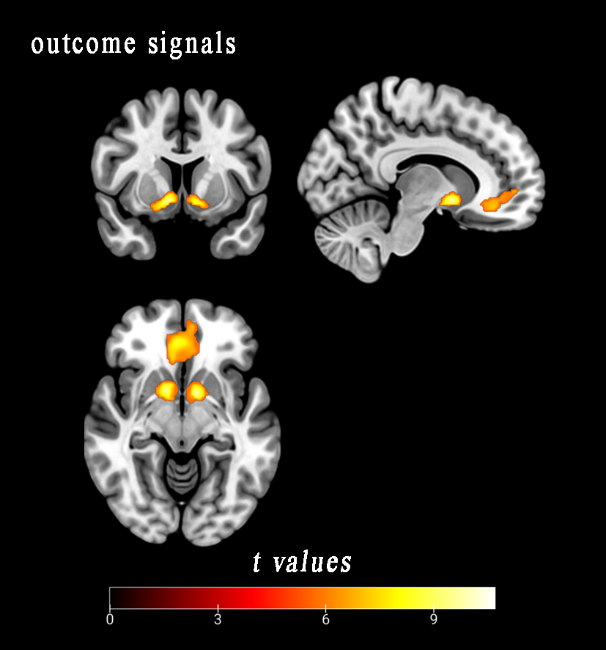

Supplement: S14 Fig — All activations were whole-brain corrected with the threshold of p < 0.001 at the voxel level and with the threshold of p < 0.05 at the cluster level using family-wise error (FWE) procedure. The source data can be found at https://osf.io/avhne/. (TIF) [file pbio.3001724.s019.tif]

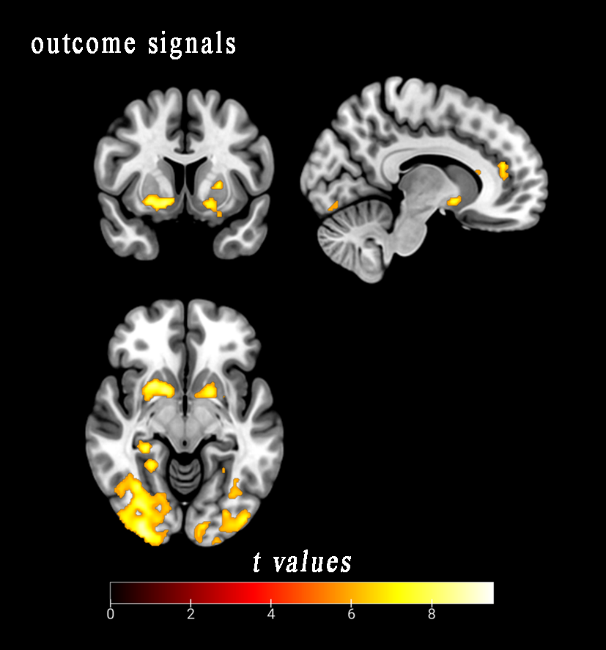

Supplement: S15 Fig — All activations were whole-brain corrected with the threshold of p < 0.001 at the voxel level and with the threshold of p < 0.05 at the cluster level using family-wise error (FWE) procedure. The source data can be found at https://osf.io/avhne/. (TIF) [file pbio.3001724.s020.tif]

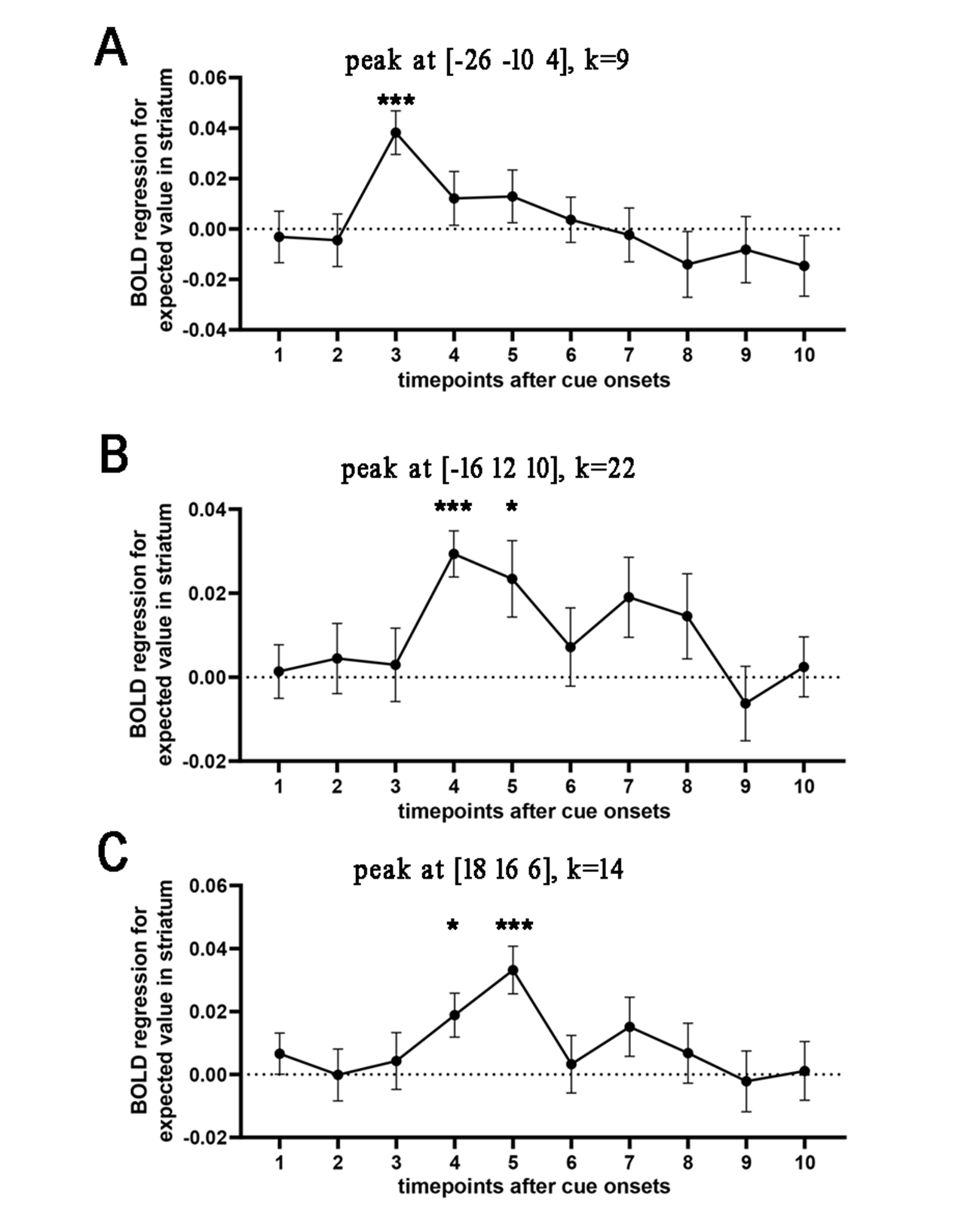

Supplement: S16 Fig — Data are represented as mean (SE). Note: ***p < 0.001; *p < 0.05. The source data can be found at https://osf.io/avhne/. (TIF) [file pbio.3001724.s021.tif]

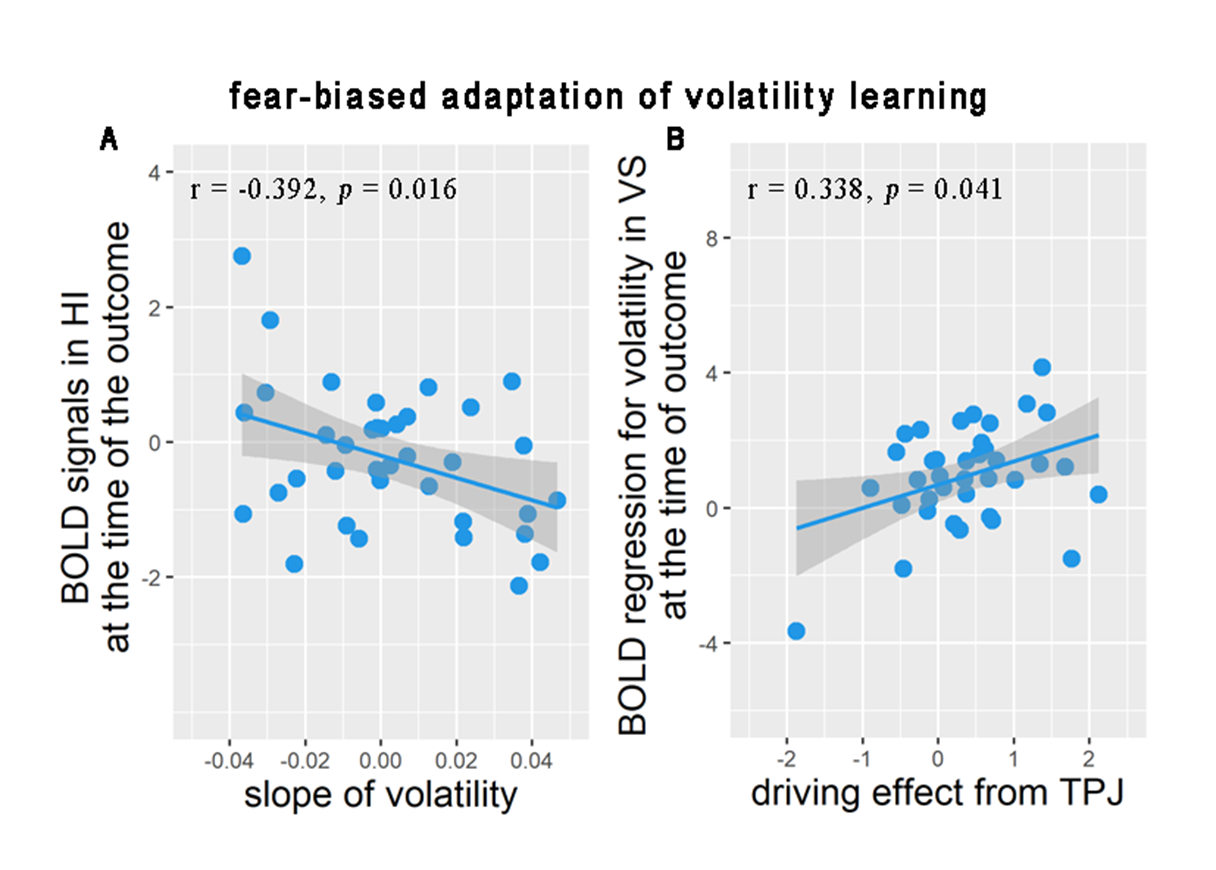

Supplement: S17 Fig — The source data can be found at https://osf.io/avhne/. (TIF) [file pbio.3001724.s022.tif]
