# Supplementary material for: Neurocomputational mechanisms underlying fear-biased adaptation learning in changing environments
Source: PLoS Biol. 2023 May 1;21(5):e3001724. doi: 10.1371/journal.pbio.3001724 (PMC10174591; doi:10.1371/journal.pbio.3001724)
Supplement: S1 Table — (DOCX) [file pbio.3001724.s023.docx]

**Table S1.** Normative rating of fearful and neutral facial expressions from the Taiwanese Facial Expression Image Database (TFEID).

| Gender | Model | Category | Correction rate (%) | Intensity (0-8) |
| --- | --- | --- | --- | --- |
| Female | F05 | Neutral | 99.1 | 0.42 |
|  | F08 | Neutral | 100.0 | 0.41 |
|  | F04 | Fear | 86.0 | 4.85 |
|  | F18 | Fear | 73.9 | 5.12 |
|  | F02 | Happy | 100.0 | 4.83 |
|  | F16 | Happy | 100.0 | 5.13 |
|  |  |  |  |  |
| Male | M04 | Neutral | 97.3 | 0.46 |
|  | M12 | Neutral | 98.2 | 0.27 |
|  | M05 | Fear | 81.0 | 5.19 |
|  | M08 | Fear | 75.9 | 5.83 |
|  | M13 | Happy | 100.0 | 5.35 |
|  | M15 | Happy | 100.0 | 5.20 |
