# Supplementary material for: Neurocomputational mechanisms underlying fear-biased adaptation learning in changing environments
Source: PLoS Biol. 2023 May 1;21(5):e3001724. doi: 10.1371/journal.pbio.3001724 (PMC10174591; doi:10.1371/journal.pbio.3001724)
Supplement: S2 Table — (DOCX) [file pbio.3001724.s024.docx]

**Table S2.** Descriptive data of post-ratings and task performance.

|  | exp1 (n = 21) | | exp2 (n = 40) | |
| --- | --- | --- | --- | --- |
|  | Mean (SD) | Range [min,max] | Mean (SD) | Range [min,max] |
| Identification rating (fear) | 6.40 (1.15) | [4,8] | 6.16 (1.65) | [2,8] |
| Identification rating (neutral) | 6.64 (1.17) | [3,8] | 6.80 (1.06) | [4,8] |
| Number of missing trials | 1.76 (1.84) | [0,6] | 2.33 (3.52) | [0,14] |
| Ratio (%) of short response time (<200 ms) | 0. 2 (0. 6) | [0, 2.5] | 0.07 (0.28) | [0,1.67] |
