# Supplementary material for: Neurocomputational mechanisms underlying fear-biased adaptation learning in changing environments
Source: PLoS Biol. 2023 May 1;21(5):e3001724. doi: 10.1371/journal.pbio.3001724 (PMC10174591; doi:10.1371/journal.pbio.3001724)
Supplement: S3 Table — (DOCX) [file pbio.3001724.s025.docx]

**Table S3.** Model recovery: model comparison for simulated data form M1

| Models | Number of parameters | exp1 (n = 21) | | exp2 (n = 40) | |
| --- | --- | --- | --- | --- | --- |
|  |  | ΔLOOIC | ΔWAIC | ΔLOOIC | ΔWAIC |
| M1 | 8 | 0 | 0 | 0 | 0 |
| M2 | 4 | 31.4 | 30.4 | 125.1 | 136 |
| M3 | 5 | -20.1 | -14.1 | 20.7 | 26.6 |
| M4 | 8 | 525.0 | 522.9 | 633.8 | 640.8 |
| M5 | 9 | -4.6 | 2.3 | -3.6 | 3.9 |
| M6 | 9 | 95.3 | 104.5 | 257.0 | 263.7 |
| M7 | 10 | 102.8 | 102.4 | 263.0 | 263.6 |
| M8 | 10 | 96.2 | 100.0 | 255.4 | 262.2 |
| M9 | 7 | 520.5 | 528.2 | 632.8 | 647.2 |
| M10 | 8 | 525.5 | 534.1 | 628.5 | 640.3 |
| M11 | 8 | 526.5 | 533.6 | 624.3 | 636.3 |
| M12 | 11 | 50.0 | 68.2 | 33.4 | 61.4 |

Abbreviations: ΔLOOIC, leave-one-out information criterion relative to the winning model; ΔWAIC, widely applicable information criterion relative to the winning model.
