# Supplementary material for: Neurocomputational mechanisms underlying fear-biased adaptation learning in changing environments
Source: PLoS Biol. 2023 May 1;21(5):e3001724. doi: 10.1371/journal.pbio.3001724 (PMC10174591; doi:10.1371/journal.pbio.3001724)
Supplement: S4 Table — (DOCX) [file pbio.3001724.s026.docx]

**Table S4.** Model comparison for expS1.

| Models | Number of parameters | exp S1 (n = 27) | |
| --- | --- | --- | --- |
|  |  | ΔLOOIC | ΔWAIC |
| M1 | 8 | 0 | 0 |
| M2 | 4 | -0.2 | 0.7 |
| M3 | 5 | -12.0 | 5.6 |
| M4 | 8 | 0.3 | -5.5 |
| M5 | 9 | -24.5 | -10.5 |
| M6 | 9 | 73.9 | 259.9 |
| M7 | 10 | 48.4 | 34.5 |
| M8 | 10 | 57.9 | 90.5 |
| M9 | 7 | -13.8 | -13.2 |
| M10 | 8 | 57.1 | 213.5 |
| M11 | 8 | -16.8 | -16.8 |
| M12 | 11 | -22.2 | -25.9 |

The winning model in expS1 is M12. Abbreviations: ΔLOOIC, leave-one-out information criterion relative to the winning model; ΔWAIC, widely applicable information criterion relative to the winning model.
