# Supplementary material for: Neurocomputational mechanisms underlying fear-biased adaptation learning in changing environments
Source: PLoS Biol. 2023 May 1;21(5):e3001724. doi: 10.1371/journal.pbio.3001724 (PMC10174591; doi:10.1371/journal.pbio.3001724)
Supplement: S5 Table — (DOCX) [file pbio.3001724.s027.docx]

**Table S5.** Model comparison for expS2.

| Models | Number of parameters | exp S2 (n = 39) | |
| --- | --- | --- | --- |
|  |  | ΔLOOIC | ΔWAIC |
| M1 | 8 | 0 | 0 |
| M2 | 4 | 739.4 | 4339.8 |
| M3 | 5 | 2.4 | 17.8 |
| M4 | 8 | 191.4 | 203.1 |
| M5 | 9 | -4.2 | 91.1 |
| M6 | 9 | 149.0 | 186.8 |
| M7 | 10 | 143.5 | 165.6 |
| M8 | 10 | 499.2 | 1791.8 |
| M9 | 7 | 162.8 | 187.7 |
| M10 | 8 | 180.8 | 213.6 |
| M11 | 8 | 177.4 | 206.9 |
| M12 | 11 | 167.1 | 488.4 |

The winning model in expS2 is M1. Abbreviations: ΔLOOIC, leave-one-out information criterion relative to the winning model; ΔWAIC, widely applicable information criterion relative to the winning model.
