# Supplementary material for: Neurocomputational mechanisms underlying fear-biased adaptation learning in changing environments
Source: PLoS Biol. 2023 May 1;21(5):e3001724. doi: 10.1371/journal.pbio.3001724 (PMC10174591; doi:10.1371/journal.pbio.3001724)
Supplement: S6 Table — (DOCX) [file pbio.3001724.s028.docx]

**Table S6.** Model comparison between the winning model (M1) and M13 assuming no effect of volatility (a learning rate and a decision parameter for each type of cue)

| Models | Number of parameters | exp1 (n = 21) | | exp2 (n = 40) | |
| --- | --- | --- | --- | --- | --- |
|  |  | ΔLOOIC | ΔWAIC | ΔLOOIC | ΔWAIC |
| M1 | 8 | 0 | 0 | 0 | 0 |
| M13 | 4 | 2.8 | 9.9 | 104.6 | 116.5 |

Abbreviations: ΔLOOIC, leave-one-out information criterion relative to the winning model; ΔWAIC, widely applicable information criterion relative to the winning model.
