# Supplementary material for: Neurocomputational mechanisms underlying fear-biased adaptation learning in changing environments
Source: PLoS Biol. 2023 May 1;21(5):e3001724. doi: 10.1371/journal.pbio.3001724 (PMC10174591; doi:10.1371/journal.pbio.3001724)
Supplement: S7 Table — (DOCX) [file pbio.3001724.s029.docx]

**Table S7**. Behavioral and BOLD responses in each experimental condition.

| Variables | fear & freq | fear && infreq | neut & freq | neut & infreq |
| --- | --- | --- | --- | --- |
| learning rate (exp1) | 0.546 (0.239) | 0.654 (0.050) | 0.695 (0.104) | 0.541 (0.223) |
| learning rate (exp2) | 0.488 (0.242) | 0.562 (0.233) | 0.644 (0.198) | 0.463 (0.226) |
| volatility activation in VS | 0.313 (1.578) | -0.117 (1.342) | -0.201 (1.210) | 0.324 (1.015) |
| volatility activation in dACC | 0.408 (0.393) | -0.158 (2.214) | -0.915 (1.860) | 0.217 (2.331) |
| FC between dACC and AG | 0.477 (2.183) | -0.385 (1.622) | -0.490 (1.572) | 0.819 (1.600) |
| driving effect on AG | 0.093 (0.552) | 0.061 (0.488) | -0.286 (0.445) | 0.061 (0.488) |

Note: Descriptive data are presented as mean (SD).
